# Genomic prediction in a small barley population can benefit from training on related populations

**DOI:** 10.1093/g3journal/jkaf218

**Published:** 2025-10-23

**Authors:** Cathrine Kiel Skovbjerg, Pernille Sarup, Ellen Margrethe Wahlström, Jens Due Jensen, Lotte Olesen, Jihad Orabi, Just Jensen, Guillaume P Ramstein, Ahmed Jahoor

**Affiliations:** Nordic Seed A/S, Kornmarken 1, Galten 8464, Denmark; Center for Quantitative Genetics and Genomics, Aarhus University, C.F. Møllers Allé 3, Aarhus C 8000, Denmark; Nordic Seed A/S, Kornmarken 1, Galten 8464, Denmark; Nordic Seed A/S, Kornmarken 1, Galten 8464, Denmark; Nordic Seed A/S, Kornmarken 1, Galten 8464, Denmark; Nordic Seed A/S, Kornmarken 1, Galten 8464, Denmark; Nordic Seed A/S, Kornmarken 1, Galten 8464, Denmark; Center for Quantitative Genetics and Genomics, Aarhus University, C.F. Møllers Allé 3, Aarhus C 8000, Denmark; Center for Quantitative Genetics and Genomics, Aarhus University, C.F. Møllers Allé 3, Aarhus C 8000, Denmark; Nordic Seed A/S, Kornmarken 1, Galten 8464, Denmark

**Keywords:** multipopulation genomic prediction, genomic selection, cross-population genomic prediction, GBLUP, genetic relationships, historical data

## Abstract

Genomic prediction (GP) has shown to be a valuable tool for genetic improvement in breeding programs but requires large training populations in order to build robust models. This is difficult to obtain for newly established breeding programs. Here, we aimed to overcome this challenge by combining datasets from 4 different barley breeding programs, utilizing up to 12 years of data to increase prediction accuracy in a more recently established 6-rowed winter (6RW) barley breeding program. By allowing data to accumulate in a breeding program as the years progress, we investigated when GP accuracy in 6RW benefitted from external populations. To do this, we focused on several parameters: training population size, choice of model for multipopulation GP (univariate versus multivariate), the key trait under investigation (grain yield, plant height, or rust resistance), and genetic distance between populations. We found that in the early stages of a breeding program, prediction of the 6RW population could benefit from inclusion of an external population, but the advantage depended on the specific population and trait under investigation. However, when data from all 4 years were available, multipopulation GP generally performed similarly to within-population GP. Additionally, when comparing multivariate and univariate models for multipopulation GP, the multivariate model often performed significantly worse, despite strong genetic correlations between the populations involved. This was especially the case when data were sparse and the model required estimation of numerous parameters from a small number of observations. Altogether, our results suggest that multipopulation GP is beneficial only in the very early stages of new breeding programs, emphasizing its relevance for newly established breeding programs or new breeding goals, especially for related populations.

## Graphical Abstract

**Graphical Abstract:**
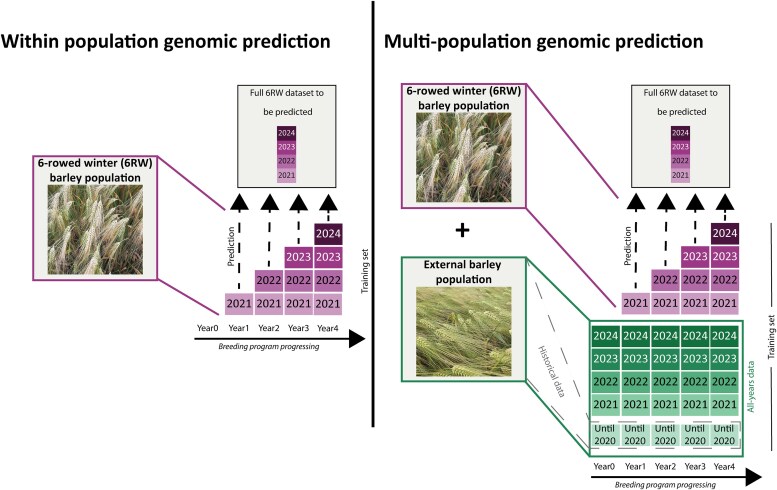

## Introduction

Genomic prediction (GP), as introduced by Meuwissen et al. (2001), utilizes genome-wide marker information to assign individuals an overall genetic merit for a trait of interest. By considering all genetic markers, no matter their effect sizes, GP has become an important tool for improving many agriculturally important traits, which often have a complex inheritance. In addition to being controlled by many small-effect loci, complex traits are influenced by environment, gene-by-gene interactions, and the interactions between genes and environments. Hence, such traits can be difficult to efficiently improve through the use of marker-assisted selection or recurrent phenotypic selection alone (Bernardo 2008; Arruda et al. 2016). With decreasing costs of genotyping, GP has become routinely implemented in plant breeding programs (Patel et al. 2015). Barley (*Hordeum vulgare* L.), one of the most widely cultivated crops worldwide, with a total production of 146 million tons in 2023 (FAO 2024), is no exception. In typical barley breeding schemes, new lines are developed from biparental crosses and made homozygous through double-haploid technology or successive selfing. Following initial visual selection by the breeder, a subset of lines enters preliminary yield trials that consist of small, unreplicated plots. At this stage, lines are genotyped, and GP is applied to select candidates for advanced testing. As lines progress through multienvironment advanced yield trials, GP is updated with new phenotypic data, and the set of lines is further narrowed down to select the best possible candidates for official registration. GP therefore supports selection decisions through the breeding pipeline, especially in case of incomplete phenotypic data or low-precision early trials (Robertsen et al. 2019).

The genetic gain achievable through breeding efforts using GP relies highly on the accuracy of predicted breeding values, which depends on several factors, one of the most important being the size of the training data used to develop the prediction model (VanRaden et al. 2009; Zhong et al. 2009; Asoro et al. 2011; Nielsen et al. 2016). In newly established breeding programs, however, limited phenotypic and genetic data usually are available, making it difficult to build sufficiently large training sets for GP. One strategy to increase the training set size is to incorporate other populations. While this approach has been investigated in studies across diverse species, the success has been highly variable. In maize, Technow et al. (2013) found that combining 2 genetically distinct small populations of dent and flint lines in the training set increased prediction accuracy for both populations. However, their study was advantaged by both populations being grown in the same field. This likely reduces phenotypic heterogeneity between populations, focusing the observed differences in performance to genetic effects and interactions between genotypes and environment. In line with this, Jarquin et al. (2016) also found that combining training populations can benefit GP in soybean. However, for optimal results, it is crucial that the geographical locations of the validation set are represented in the training set, especially when the geographical locations are significantly different.

Another factor that influences prediction accuracy is the genetic relationship between training and validation sets. Previous studies suggest that multipopulation GP is most effective when the genetic correlation between populations is moderate to high (Technow et al. 2013; Raymond et al. 2020) and the size of the target population is small (Technow and Totir 2015). In crops, some studies have even suggested that increasing the training population with increasingly dissimilar individuals (compared to the validation set) may reduce the overall prediction ability (Lorenz et al. 2012; Adeyemo et al. 2023), whereas other studies in crops have found that adding distantly related individuals can still benefit or at least not harm the prediction accuracy (Technow et al. 2013; Jarquin et al. 2016; Sverrisdóttir et al. 2018). A less extreme case of combining distinct populations occurs when utilizing historical data of a population. Increasing a training set with historically older lines is common practice in GP for applied breeding programs, where training sets consist of lines from previous years. In oat, Asoro et al. (2011) found that adding older lines to training populations either increased or did not affect the prediction accuracy of new lines.

When considering genetically distinct populations in plant breeding, the concept of breeds in animals provides a useful analogy. In cattle, studies on multibreed GP have also found that a target population can benefit from including other populations into the training set. Here, many different approaches have been tested, and it has continuously been demonstrated that success relies heavily on single-nucleotide polymorphisms (SNPs) that are in linkage disequilibrium (LD) with quantitative trait loci (QTLs) in both breeds—secured through either high marker density or methods that allow for extra weighting of such SNPs (Hayes et al. 2009; Erbe et al. 2012; Raymond et al. 2018, 2020). In general, the choice of model is important when combining populations. While most approaches for combining populations in crops have relied on a simple univariate model that assumes constant marker effects across populations (i.e. for the same trait measured in different populations, the genetic correlation is assumed to be 1), multiple studies in cattle have shown that a multivariate GP model allowing marker effects to be different between populations typically works better (De Haas et al. 2012; Olson et al. 2012; Wientjes et al. 2016). Since multipopulation GP models are inherently more complex and computationally demanding than simple within-population GP models, it is important to carefully consider in which scenarios the benefits of multipopulation GP outweigh the drawbacks. Lehermeier et al. (2015) evaluated 3 GP strategies: (1) within-population prediction, (2) multipopulation prediction ignoring genetic heterogeneity (i.e. assuming that the genetic architecture of a trait is the same across populations), and (3) multipopulation prediction that accounts for genetic heterogeneity by modeling population-specific genetic variance while allowing for variable genetic correlation between populations. They found that the choice of method most suitable for GP depended on the genetic distance between the combined populations, the trait of interest, and the sample size. Although the differences in model performance were typically relatively small, the greatest advantage of modeling genetic heterogeneity when combining populations appeared when sample sizes were large and populations exhibited some degree of differentiation. On the other hand, if populations showed such a high degree of genetic heterogeneity that genetic correlations were close to 0, within-population prediction could perform just as well as multipopulation models that account for genetic heterogeneity. However, even in such cases, multipopulation models that account for genetic heterogeneity still outperformed multipopulation approaches ignoring the genetic heterogeneity of different populations (Lehermeier et al. 2015). In general, one would expect multipopulation GP to be useful in improving prediction accuracy when the *true* genetic correlation between the same trait scored in different populations is different from 0 and estimated with little error. In barley, multipopulation GP has been applied on several occasions, and full datasets of different breeding programs have been combined to create 1 large training set that was hypothesized to improve the overall prediction accuracy (Lorenz et al. 2012; Ankamah-Yeboah et al. 2020). While this has rarely been proven effective, little has been done to comprehensively study the circumstances under which a progressing breeding program can benefit from inclusion of an external population.

In this study, we focus on a newly established 6-rowed winter (6RW) barley breeding program at Nordic Seed A/S. The objective of this study was to understand the circumstances under which GP of this small 6RW barley population can be improved by incorporating data from other parallel barley breeding programs. By integrating data on grain yield, plant height, and rust resistance from the 6RW population with data from 3 other well-established breeding populations with up to 12 years of data, we seek to answer how prediction accuracy using a multipopulation GP approach is influenced by (1) the genetic relationship between the 6RW population and the external population in the training set, (2) the sample size and/or number of training years for the 6RW population, and (3) the choice of model for combining observations across different populations.

## Materials and methods

### Plant material and genotyping

The genetic material used in this study consisted of 30,427 barley lines representing 4 distinct breeding populations maintained by Nordic Seed A/S. These included 2,513 6RW lines, of which 619 were phenotyped; 6,364 two-rowed winter (2RW) lines, of which 1,753 were phenotyped; 1,349 six-rowed spring (6RS) lines, of which 658 were phenotyped; and 20,201 two-rowed spring (2RS) lines, of which 4,212 were phenotyped. All lines were inbred to at least F5 (spring types) or produced using double-haploid technology (winter types).

Our target population in this study is the 6RW program. It was established in 2017 to support the development of hybrid barley varieties. At the time of analyses (2024), the 6RW population consisted of parental components of a hybrid breeding program: 134 phenotyped restorer lines and 485 phenotyped maintainer lines referred to as R- and B-lines, respectively. The 6RS program was initiated in 2015 to meet the demands for early-maturing feed barley varieties adapted to the Nordic and Baltic regions. The 2RW program was established in 2011 as a European feed barley program. The 2RS is the oldest of the 4 breeding programs and was established in 1935 with a focus on developing malting and feed barley for the European market. The breeding programs are managed as separate populations and are rarely interbred. More details on the breeding populations can be found in Skovbjerg et al. (2025).

Initially, the SNP dataset comprised 13,722 markers genotyped across 30,427 individuals from all 4 barley populations. This dataset was filtered for missingness (retaining individuals with ≤20% missing genotypes and markers with ≤20% missingness) and duplicate positions. It was then imputed within each population using Beagle v.5.4 (Browning et al. 2018) as described in Skovbjerg et al. (2025). Although many of the 30,427 individuals did not have phenotypic records, this large dataset was used to leverage all available genotype data for imputation. Following this initial processing, 13,555 markers remained. To ensure a consistent set of markers across all populations and GP scenarios, we applied a minor allele count (MAC) filter across all populations, retaining only markers with a MAC of at least 20 across all 4 populations. This resulted in a final dataset of 13,035 SNPs used for downstream analyses. The quality control workflow is summarized in Supplementary Fig. 1.

### Evaluation of genetic redundancy, relationships, and population structure

To identify population outliers, we performed principal component analysis (PCA) on all lines from the 4 populations (*n* = 30,427) using PLINK v.1.9 (Purcell et al. 2007). Based on the first 3 principal components (PCs), we identified a total of 13 lines that were positioned closer to the median of a different population's cluster along PCs 1-3 than to the population they were originally assigned to. These 13 lines were excluded from further analyses.

To prevent bias from near-identical genotypes appearing in both the training and validation sets in GP, we assessed each population for genetic redundancy. If a set of lines differed by <100 SNP alleles, they were considered replicates of the same genotype. In such cases, the oldest line was interpreted as the “true line.” Phenotypic observations from redundant lines were reassigned to their “true line” by renaming them in the phenotype file, while the redundant lines had their SNP data removed from the genotype dataset to ensure each genotype was represented only once. The number of lines renamed due to genetic redundancy was 31 (4.8%), 19 (1.1%), 1 (0.2%), and 121 (2.8%) in 6RW, 2RW, 6RS, and 2RS, respectively (see Supplementary Fig. 2 for distributions of pairwise SNP differences). Of the 30,427 genotyped lines, a total of 7,242 also had phenotypic data available. All analyses beyond quality control, including visualization of population structure, mixed models, and GP, were performed using this phenotyped and genotyped subset.

Further, the genetic relationship between the 6RW populations and another population was assessed using the pairwise off-diagonal elements of the genomic relation matrix (**G**) including both populations. The **G** matrix used for modeling was calculated following the approach outlined in VanRaden (2008):


(1) $$G=\;\frac{ZZ^{T}}{2\sum_{i}p_{i}(1-p_{i})}$$

where **Z** is a matrix of centered genotypes with dimension *n* × *m*, where *n* refers to the number of individuals and *m* refers to the number of markers. *p_i_* denotes the allele frequency at SNP *i* across the involved populations. To ensure a consistent comparison of pairwise relationship coefficients within and between populations, the **G** matrix used for the genetic relationship density plots was modified by using the allele frequencies in the 6RW population exclusively in the denominator of Equation (1).

The per-SNP fixation index (*F*_ST_) was calculated using VCFtools to assess genetic differentiation between populations at each SNP (Danecek et al. 2011).

### Field experiments and phenotyping

Grain yield, plant height, and rust resistance were scored in multiple combinations of years and locations, referred to as environments. In total, phenotypic data included scorings from 2013 to 2024 from a set of 3 locations in Denmark and 1 in Germany. The Danish fields were located in Skive (56°37′41.3″N 9°02′35.6″E), Odder (55°57′19.5″N 10°14′26.0″E), and Holeby (54°42′41.6″N 11°27′59.4″E). The German location was in Nienstädt (52°17′36.6″N 9°08′53.6″E). Grain yield was scored in more environments than plant height and rust resistance in most populations and was generally available wherever the other traits were assessed. However, there are a few exceptions where grain yield data are missing, despite the availability of data for the other traits (e.g. Odder in 2021 and 2024 for 6RW), because the field was not harvestable, mostly due to weather conditions (Supplementary Table 1).

In the 6RW population, grain yield, plant height, and rust resistance were scored in 12, 8, and 4 environments, respectively. For the remaining populations, grain yield was scored in 37 (2RW), 4 (6RS), and 40 (2RS) environments, and plant height were scored in 18 (2RW), 6 (6RS), and 10 (2RS) environments, while rust resistance were scored in 9 (2RW), 6 (6RS), and 11 (2RS) environments. The data for all populations were restricted to observations from locations shared with 6RW. This was done in order to limit genotype × environment interactions (GxE). Supplementary Table 1 provides a list of the environments along with the corresponding number of observations per population and trait. In the fields where grain yield and plant height were scored, the plot sizes varied between locations but were between 8.25 and 9.75 m^2^. Disease resistance was scored in Seedmatic field trials where plot sizes were ∼1 m^2^. Fungicides were applied in the fields where grain yield and plant height were scored, but not in the fields where rust resistance was scored. In general, fields were managed according to the best management practices for conventional farming.

For each year, genotypes were organized into trials and tested across multiple locations using an alpha-lattice design (5 lines per miniblock), with 2 to 3 replicates. The average number of lines per trial was 38.9 in 6RW, 34.5 in 2RW, 56.3 in 6RS, and 23.4 in 2RS. In 2RS, trial information was lacking for disease data. As traits were routinely assessed in advanced breeding programs where a new set of crosses was produced every year, the set of phenotyped lines varied from year to year with some degree of overlap between adjacent years (Supplementary Figs. 3 to 14).

The traits were scored in the following way:

1. Grain yield was measured in hectokilogram (hkg) per hectare (ha), adjusted for 15% moisture content. Outlier phenotypes with grain yields >200 hkg ha^−1^ were removed.
2. The plant height (cm) was measured in each plot by selecting a representative plant and measuring the distance from the base of the plant to the top of the spike, excluding awns. Measurements were taken at maximum height, approximately at stage 65 on the BBCH scale (Biologische Bundesanstalt, Bundessortenamt und CHemical Industry), i.e. when 50% of the anthers were mature (SEGES Innovation 2023).
3. Rust resistance was assessed 1 to 2 times within each field using a scale from 1 (resistance) to 9 (very severe infection) (see Supplementary Table 2). The first assessment was done at disease onset, and the second ∼1 week later, both during BBCH stages 32 to 59, i.e. from when nodes became visible to right before flowering began (SEGES Innovation 2023). If rust resistance was scored on 2 dates within a field, data were averaged over values, which corresponded to the area under the disease progress curve.

### GP models

GP was performed within the 6RW population and by multipopulation GP where a distinct barley population, 2RW, 6RS, or 2RS, was included in the training set used for fitting the model. The models applied in this study are modified versions of the models described in Skovbjerg et al. (2025). For prediction within the 6RW population, the applied model can be written as follows:


(2) $$y\;=X_{1}\mu +\;X_{2}l+X_{3}h+Z_{1}g_{a}+Z_{2}g_{l}+Z_{3}w\;+Z_{4}v\;+\overset{15}{\underset{\;j=1}{\sum }}\;Q_{j}s+e\;$$

Here, **y** is a vector containing the observed phenotypes; *µ* is the intercept of the model; **l** is a vector containing the fixed effects associated with year × location × trial, where trial is nested within population; **h** is a vector of the fixed effects associated with the different heterotic groups (B or R); **g**_a_ is a vector of SNP-based genomic breeding values for lines with $g_{a}∼N(0,G\sigma_{ga}^{2})$, where **G** is the genomic relationship matrix and $\sigma_{ga}^{2}$ is the additive genetic variance; **g**_l_ is a vector containing the residual line effects that is not captured by the additive marker effects in $g_{a}$, $g_{l}∼N(0,I\;\sigma_{gl}^{2})$; **w** is a vector of additive genetics × environment interaction effects with $w∼N(0,I⊗G\sigma_{w}^{2})$, where environments are nested within population; **v** is a vector of nonadditive genetics × environment interaction effects with $v∼N(0,I\sigma_{v}^{2})$; ***s*** is a vector of spatial effects within the field where $s∼N(0,I\sigma_{s}^{2}).\;$ The spatial effects (**s**) are modeled as a moving window containing the plot itself and the 14 surrounding plots (Skovbjerg et al. 2025). Plots located at the edge of a field had fewer real neighbors, so the missing neighbors were set to empty plots to preserve the structure of the moving window. For rust resistance, the spatial location of the plots within field was unknown, and therefore, spatial effects were not included. **e** is a vector of residual effects where $e∼N(0,I\sigma_{e}^{2})$. **X_1_**, **X_2,_** and **X_3_** are the design matrices for the fixed effects. **Z_1_**, **Z_2_**, **Z_3_**, **Z_4_**, and **Q_j_** are the design matrices for the random effects. Equation (2) was also applied within each population (6RW, 6RS, 2RW, 2RS) in order to estimate variance components and trait heritability. Before displaying the variance components in a percent stacked bar plot, the estimated values of $\sigma_{ga}^{2}$ and $\sigma_{w}^{2}$ were multiplied by the mean diagonal of the **G** matrix to account for inbreeding. Similarly, the spatial variance was multiplied by 15 to account for the contribution of 15 plots within the moving window.

For the multipopulation prediction, 2 different models were applied. The first model, hereafter referred to as MP1, was a univariate multipopulation model that assumes that the effect of each marker is the same across different populations, thus ignoring potential genetic heterogeneity. The second model, MP2, was a multivariate multipopulation model that allowed marker effects to differ between populations while considering correlations between them.

The MP1 model was identical to the model presented in Equation (2), but to account for different environmental effects experienced by different populations, the environmental (**l**) and spatial effects (**s**) were nested within populations. For spatial effects, a common variance was estimated across both populations. For 2RS individuals, the *y*-coordinates of the field plots were unknown for all traits. For this reason, the spatial effect (**s**) of grain yield and plant height is modeled as a random effect of the *x*-coordinate within the field. In addition, trial information was unavailable for rust resistance in the 2RS population. As a result, the fixed effect of year × location × trial was reduced to a year × location effect. As no heterotic groups are present in the 2RW, 6RS, or 2RS population, all individuals from these populations are set to belong to the same heterotic group, which is different from the B and R groups.

The MP2 model was a multivariate version of the described univariate model. For the additive genetic variances $\left(g_{a*}\right)$, it follows that


$$g_{a*}∼MVN(0,\Sigma_{ga*}⊗G)$$

where the variance–covariance matrix $\left(\Sigma_{ga*}\right)$ is as follows:


$$\Sigma_{ga*}=\left[\begin{array}{cc} \sigma_{ga,pop1}^{2} & \sigma_{ga,pop1,2}^{2}\\ \sigma_{ga,pop1,2}^{2} & \sigma_{ga,pop2}^{2} \end{array}\right]$$

The modeling of genetic covariance allowed for estimating the genetic correlation of the same trait scored in different populations. MP2 also relaxed the assumption of common variance across populations, resulting in twice the number of variance components in MP2 compared to MP1 in addition to the genomic covariance parameter. In MP2, all variance components (genetic variance, GxE variance, spatial variance, and residual variance) were allowed to differ between populations. For additive genetic effects, this includes separate variances for each population as well as their genetic covariance, resulting in 3 (co)variance parameters instead of 1 in MP1. No covariances were modeled for the remaining components, which were treated as independent across populations. While some environments share location–year labels (e.g. OD:2024), we treated environments as population specific due to the different growing seasons of winter and spring barley. Including cross-population GxE covariances in the model would increase its complexity substantially and would most likely not be well estimated due to the size of our dataset. Since our main objective is to predict performance across environments rather than model GxE structure in detail, we did not include such covariances.

All GP and variance component estimation were performed using the software DMU (Madsen and Jensen 2013).

### Cross-validation

Different cross-validation (CV) approaches were employed in this study; all aimed at evaluating the predictive ability of the different genomic models for all 6RW lines. When a CV scheme included only 6RW lines in the training population, it was referred to as a “within-population” prediction approach. In contrast, “multipopulation prediction” described cases where the training population consisted either entirely of a different population than 6RW or a mix of 6RW individuals and other populations.

The CV schemes were set up to mimic the cultivar development process where training data accumulate up to training year *k* (*k* = 2021, 2022, 2023, 2024). For this reason, the training set consisted of 1 (2021), 2 (2021 to 2022), 3 (2021 to 2023), or 4 years of data (2021 to 2024). Thus, for evaluating line *i* in training year *k*, the training set excluded all observations from line *i* across all years as well as any observations from years later than year *k*. As a result, this meant that as the training year advanced, the training set included both more lines and observations per line.

Within each training year, the overall performance of each 6RW line, represented by a single genomic estimated breeding value (GEBV) across all environments, was predicted exactly once using 1 of 2 CV strategies depending on the line's data availability. The 2 CV strategies were (1) forward prediction for untested lines and (2) a leave-one-line-out (LOLO) approach for lines with observed phenotypes in training year *k* or earlier.

1. Forward prediction of untested lines: Lines with no phenotypic observations in training year *k* or earlier were treated as entirely new and were predicted using models trained on all available lines up to and including year *k*.
2. LOLO prediction of observed lines: For lines that had phenotypic data in training year *k* or earlier, we applied a LOLO approach. Each line was predicted by removing all its phenotypic observations across all years from the training data.

**Fig. 1.**
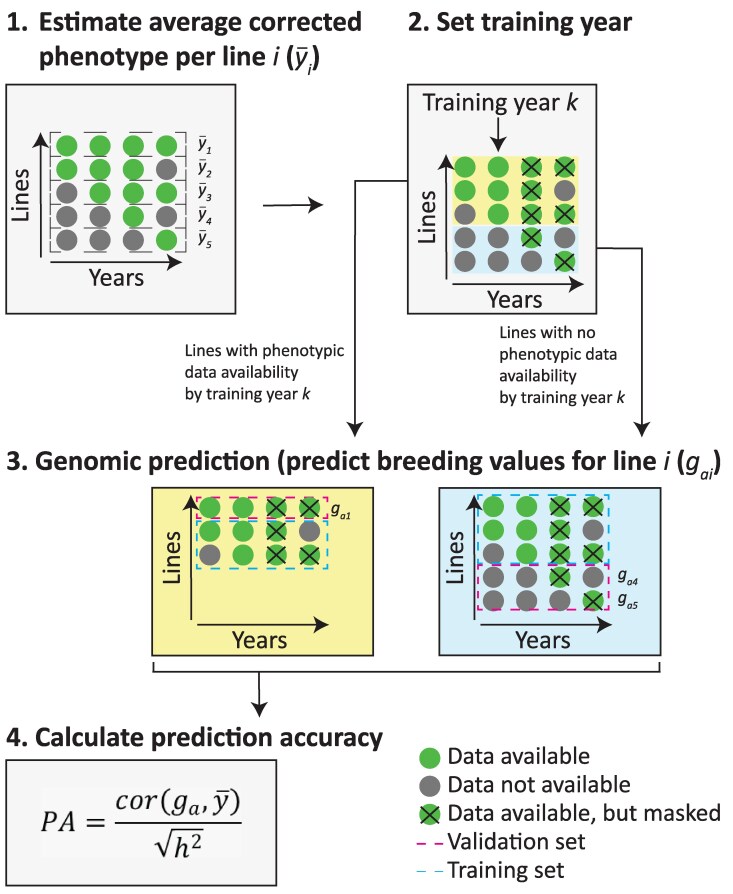
Schematic overview of the cross-validation setup, exemplified using the second year as the training year. All phenotypic data from later years are masked to mimic realistic forward prediction. Each dot represents a line-by-year combination and indicates the presence (green) or absence (gray) of phenotypic observations for that line in a given year. Blue squares mark the training set and pink squares mark the validation set. Only 5 lines are considered for illustrative purposes. The figure illustrates a within-population genomic prediction (GP) approach. For multipopulation GP, the training set includes not only a subset of 6RW lines but also the complete historical or all-years dataset from an external population.

Our CV was designed to maintain a constant number of predicted lines between training years, while allowing the size of the training population to increase over time, thereby reflecting how data accumulate in a typical breeding program. A schematic overview of the CV scheme is provided in Fig. 1.

Using a multipopulation prediction approach, the training set was expanded to include observations from lines belonging to a distinct extra population (6RS, 2RS, or 2RW). In contrast to the 6RW training data, which increased in size over different training years, the external population's training dataset remained fixed in size across all training years. This ensured that as the training year progressed, only the size of the 6RW training set increased, allowing us to clearly separate the effects of getting more 6RW training data from the impact of including an external population in the training set.

To distinguish between the effects of simply adding additional lines from the external population versus adding lines that provide additional information about years represented by the 6RW population to be predicted, we considered 2 versions of the external data. The “historical data” version included only phenotypic data collected up to and including 2020 (2015 to 2020 for 2RW, 2019 to 2020 for 6RS, and 2013 to 2020 for 2RS), thus excluding phenotypes from years overlapping with the 6RW data. In contrast, the “all-years” data version included phenotypic data from all years, including those subsequent to the training year (2015 to 2024 for 2RW, 2019 to 2024 for 6RS, and 2013 to 2024 for 2RS). In the MP1 multipopulation prediction approach, the training years were extended to year 0 (2020), where no 6RW observations were available, ensuring cross-population prediction only. The exact number of lines contributing to the training set in a given training year can be found in Table 1.

**Table 1.** Overview of size (number of lines) of training sets in the different prediction approaches.

		Individuals in training population						
Trait	Training year	6RW*^a^*	6RW + 2RW		6RW + 6RS		6RW + 2RS	
			Hist.	All.	Hist.	All.	Hist.	All.
Grain yield	2020 (0)	0	890	1,753	146	497	2,590	4,211
	2021 (1)	48	938	1,801	194	545	2,638	4,259
	2022 (2)	224	1,114	1,977	370	721	2,814	4,435
	2023 (3)	389	1,279	2,149	535	886	2,979	4,600
	2024 (4)	566	1,456	2,319	712	1,063	3,156	4,777
Plant height	2020 (0)	0	838	1,688	223	658	2,025	2,025
	2021 (1)	223	1,061	1,911	446	881	2,248	2,248
	2022 (2)	224	1,062	1,912	447	881	2,249	2,249
	2023 (3)	389	1,227	2,077	612	1,047	2,414	2,414
	2024 (4)	564	1,402	2,252	787	1,222	2,589	2,589
Rust resistance	2020 (0)	0	685	1,488	200	631	2,524	3,642
	2021 (1)	148	833	1,636	348	779	2,672	3,790
	2022 (2)	215	900	1,703	415	846	2,739	3,857
	2023 (3)	351	1,036	1,839	551	982	2,875	3,993
	2024 (4)	581	1,266	2,069	781	1,897	3,105	4,223

Hist., Historical data: All., All-years data.

^a^One line less for leave-one-out prediction of lines with the years of data we already have phenotypes on.

In addition to the described validation approach, we implemented multipopulation prediction approaches, where a progressively increasing sampled subset of 6RW lines was included in the training population. This was done to (1) determine the point at which the 6RW training population sample size became sufficient to no longer benefit from the inclusion of a distinct other population and (2) to avoid confounding of the age and size of the training population, as was observed in the forward-validation approach. The number of 6RW individuals in the training set began at 25 and was increased by increments of 25 until the full population was included. The procedure was repeated 10 times.

To improve computational efficiency, variance components were not reestimated each time a line was left out in the LOLO procedure but kept constant within each training set, i.e. within training year and the randomly sampled subsets. Our experience indicates that this does not markedly influence the GEBVs because only a single line is left out in each round of the LOLO CV.

### Prediction accuracy

The accuracy of a prediction model (PA) was defined as the Pearson correlation between the GEBVs and the mean phenotypes corrected for fixed effects:


(3) $$PA=\frac{cor({\hat{g}}_{a},\bar{y})}{\sqrt{h^{2}}}$$

where ${\hat{g}}_{a}=[{{\hat{g}}_{a}}_{1},{{\hat{g}}_{a}}_{2},\ldots ,{{\hat{g}}_{a}}_{N}]$ is the vector of GEBVs obtained through CV for all *N* lines,$\bar{y}=[{\bar{y}}_{1,}{\bar{y}}_{2},\ldots ,{\bar{y}}_{N}]$ is the vector of mean-corrected phenotypes for the same lines, and *h^2^* denotes the entry-mean narrow-sense heritability.

Each line *i* was observed across multiple environments, resulting in *n_i_* observations. Phenotypes were corrected for fixed effects as follows:


(4) $${y_{cor}}_{ijk}=y_{ijk}-\hat{\mu }-\hat{h_{i}}-\hat{l_{j}}$$

where *y_ijk_* is the *k*th raw observation of line *i* in the *j*th environment, $\hat{\mu }$ is the model intercept, $\hat{h_{i}}$ is the effect due to the heterotic group of line *i*, and $\hat{l_{j}}$ is the fixed effect of the *j*th environment. To get 1 value per line, the corrected phenotypes were averaged as follows:


(5) $${\bar{y}}_{i}=\frac{1}{n_{i}}\underset{j,k}{\sum }\;{y_{cor}}_{ijk}\;$$

The design (entry-mean) narrow-sense heritability was calculated as follows:


(6) $$h^{2}=\;\frac{\hat{\sigma_{ga}^{2}}}{\hat{\sigma_{p}^{2}}}\;\;\;\;\;\;$$

(7) $$\hat{\sigma_{p}^{2}}=\hat{\;\sigma_{ga}^{2}}+\hat{\sigma_{gl}^{2}}+\frac{\hat{\sigma_{w}^{2}}}{n_{e}}+\frac{\hat{\sigma_{v}^{2}}}{n_{e}}+\frac{\hat{\sigma_{s}^{2}}}{n_{r}}+\frac{\hat{\sigma_{e}^{2}}}{n_{r}}$$

where *n_e_* and *n_r_* indicate the average number of environments per line and the average number of observations per line across all environments, respectively.

Both the phenotypes corrected for fixed effects and the entry-mean narrow-sense heritability were estimated by applying the model described in Equation (2) to the full set of 6RW data without applying CV.

## Results

### Genetic diversity, relationships, and differentiation across populations

**Fig. 2.**
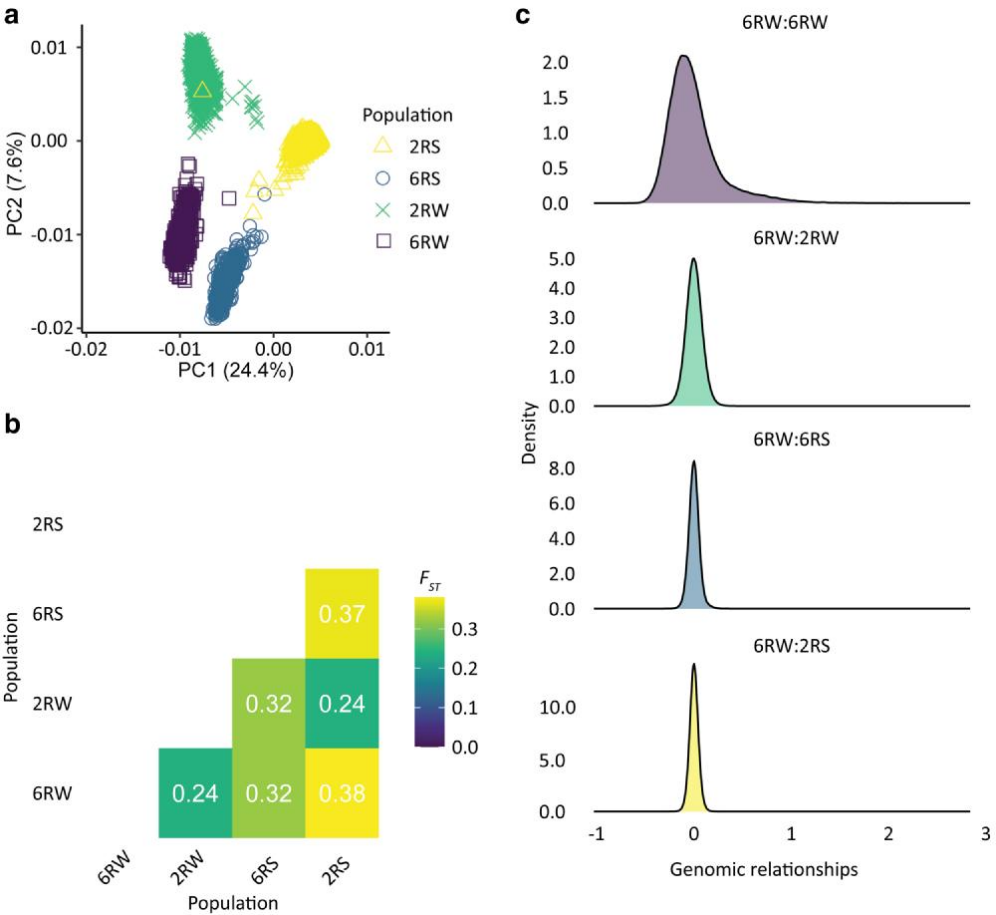
Population structure and cross-population relationships of the 4 barley populations. a) PCA plot showing the first 2 dimensions. b) A genetic differentiation matrix showing the pairwise *F*_ST_ values between populations. c) Density plots displaying the pairwise relationship coefficients from the **G** matrix between 6RW lines (purple, off-diagonals only), between 6RW and 2RW lines (green), between lines from 6RW and 6RS (blue), and between 6RW and 2RS (yellow).

In order to evaluate how genetically distinct the 4 different breeding programs were, we performed PCA and *F*_ST_ analysis and computed the **G** matrix using genotypes from all 7,242 plant lines, each genotyped for 13,035 SNP markers (Fig. 2). The first 2 PCs collectively explained 32% of the variance across SNPs and clearly separated the populations into 4 genetic clusters. As previously observed in Skovbjerg et al. (2025), PC1 separated winter versus spring types, whereas PC2 separated the breeding programs based on row type (Fig. 2a). PC3 explained an additional 5% of the variance (Supplementary Fig. 15). The *F*_ST_ values reflected the same overall pattern as the PCA plot, indicating that the 6RW population is genetically closest to the other winter-type population, 2RW (*F*_ST_ = 0.24) and genetically most differentiated from the 2RS population (*F*_ST_  *=* 0.38), which both differ in row type and growth habit. In general, the *F*_ST_ values showed moderate to strong differentiation between all pairs of populations, introducing a potential challenge in leveraging shared genetic effects across populations (Fig. 2b). The elements of the **G** matrix confirmed that genetic relationships were stronger within the 6RW population than across populations. Within 6RW, the distribution of off-diagonal pairwise relationship coefficients was skewed, with a long tail extending toward higher values, indicating the presence of very close relatives, i.e. full sibs. In contrast, relatedness between 6RW lines and lines from other populations was tightly distributed around 0, suggesting generally low cross-population genetic relationships. Although largely unrelated, the maximum relationship coefficient between 6RW and 2RW lines was high (Fig. 2c, Supplementary Table 3). When comparing 6RW and 2RW lines, the vast majority of lines showed at least some moderate cross-population relationships, with 91% of the 2RW lines and 96% of the 6RW lines exhibiting a cross-population genomic relationship coefficient of ≥0.2 to at least 1 individual. This indicates that the GP of a large proportion of the 6RW population can potentially benefit from inclusion of 2RW lines. In comparison, the relationships between individuals of 6RW and either 6RS or 2RS were much weaker, but still some relationships existed, which could be utilized for multipopulation GP. In fact, 23% of the 6RS lines and 8% of the 2RS lines had a genomic relationship coefficient of ≥0.2 with at least one 6RW line. Additionally, 23% and 18% of the 6RW lines had a genomic relationship coefficient of ≥0.2 with at least one 6RS or 2RS line, respectively.

### Trait characterization, variance components, and heritability

To investigate if the phenotypic distribution for all 3 traits also varied significantly between the populations, the overall trait distribution, average, minimum, and maximum values were compared alongside the coefficient of variation (Table 2, Supplementary Figs. 16 to 27). On average, grain yield was higher for winter types than spring types, and their coefficient of variation was higher. The highest grain yield average of 103.6 hkg ha^−1^ was observed in the 6RW population, whereas the 2RW population showed the greatest potential with a maximum value of 155.1 hkg ha^−1^. In addition, based on the datasets used in this study, winter-type plants were generally taller than spring types, with the 6RW population exhibiting an average height of 97.1 cm, while the 6RS population had the shortest plants on average, at 71.7 cm. This is likely due to the presence of the semi-dwarfing gene, *sdw1/denso*, in European spring barley to improve lodging resistance and delay heading (Skovbjerg et al. 2025). The 6RS population also showed the largest variability of plant height with a coefficient of variation at 16.7%. In general, all populations, except 6RW, showed relatively little severity of leaf rust with average values below 4, i.e. the rust covered <10% of the lower leaves. Despite the relatively high mean susceptibility of the 6RS population (3.9) compared to the 2RS (3.6) and 2RW (2.9) populations, the degree of susceptibility never reached a scale higher than 7. In contrast, the 6RW population had an average score of 4.5, indicating that all the plants were infected with 10% to 25% on lower leaves and up to 5% on top leaves. The 2RW population was the only one to have an observation of maximum severity, i.e. score of 9, and showed considerable variability, with a coefficient of variation of 58.2%.

**Table 2.** Descriptive statistics of phenotypic observations.

Trait	Population	Mean	Minimum	Maximum	Coef. Var. (%)
Grain yield (hkg ha^−1^)	6RW	103.6	33.2	145.1	16.8
	2RW	96.0	41.6	155.1	16.9
	6RS	72.4	41.3	91.3	10.1
	2RS	80.8	29.4	130.8	13.0
Plant height (cm)	6RW	97.1	70.0	130.0	10.8
	2RW	90.1	55.0	125.0	12.9
	6RS	71.7	40.0	110.0	16.7
	2RS	81.2	17.0	110.0	11.5
Rust resistance (score 1–9)	6RW	4.5	1.5	8.0	24.0
	2RW	2.9	1.0	9.0	58.2
	6RS	3.9	1.0	7.0	41.2
	2RS	3.6	1.0	8.0	35.5

Coef. Var., Coefficient of variation.

The entry-mean narrow-sense heritability was moderate to high (0.35 to 0.80) for all 3 traits across the 4 populations, although the proportion of variance explained by each model parameter varied considerably depending on population and trait. In most populations, plant height was the most heritable trait. The population-specific heritability varied from 0.35 (6RW) to 0.48 (2RW and 6RS) for grain yield, from 0.45 (6RS) to 0.80 (6RW and 2RW) for plant height, and from 0.42 (2RS) to 0.64 (6RW) for rust resistance (Table 3).

**Table 3.** Narrow-sense design heritability based on line means.

Population	*h* ^2^		
	Grain yield	Plant height	Rust resistance
6RW	0.35 (0.07)	0.80 (0.03)	0.64 (0.06)
2RW	0.48 (0.04)	0.80 (0.02)	0.52 (0.04)
6RS	0.48 (0.08)	0.45 (0.07)	0.62 (0.06)
2RS	0.46 (0.03)	0.79 (0.04)	0.42 (0.03)

The parentheses contain the standard errors of the estimate.

**Fig. 3.**
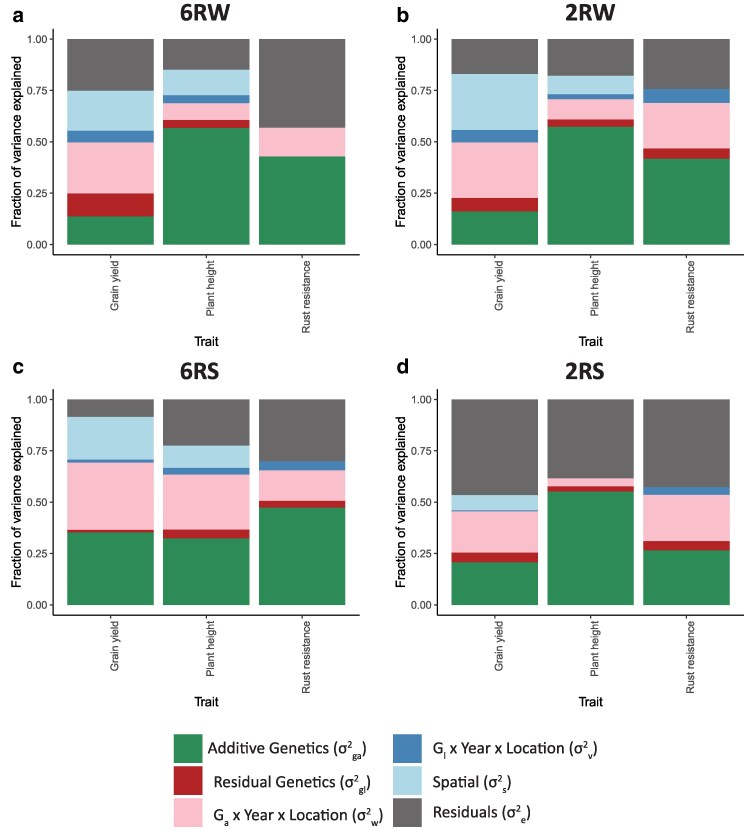
Estimated variance components across traits and populations. To account for inbreeding, the estimated values of $\sigma_{ga}^{2}$ and $\sigma_{w}^{2}$ have been multiplied by the average diagonal of the **G** matrix. Further, $\sigma_{s}^{2}$ has been multiplied by 15 to account for the contribution of 15 plots within the moving window. Note that the variance components are not directly comparable to the heritability estimates, as heritability has been calculated as design heritability on a line mean basis, whereas the variance components have not been divided by the number of observations per line or the average number of environments per line.

Figure 3 displays the distribution into variance components within individual populations. Except for grain yield and plant height when comparing 6RW with 2RW, the relative variance explained by the different parameters showed large differences across populations and traits, favoring the assumptions of the MP2 over MP1. As expected, most of the genetic variance for all traits was captured by the additive SNP information; however, in the 6RW population, a substantial amount of the overall phenotypic variance (11%) in grain yield was captured by nonadditive genetic effects or genetic effects not captured by the SNP markers (residual genetic variance). The amount of phenotypic variation explained by GxE varied significantly between populations and traits. On average, grain yield was the trait most affected by GxE, while plant height was the least affected. The proportion of variance explained by GxE varied from 4% for plant height in 2RS to 35% for grain yield in 6RS. For all traits, the 2RS population showed a large proportion of the trait variation due to residual effects. In addition, rust resistance was the trait with the largest residual variance. We suspect that the presence of substantial residual variation might be due to the lack of a sophisticated correction for spatial effects within the field, when applying models to rust resistance data or to the 2RS population. In fact, the spatial variation can explain up to 27%, as observed for grain yield in the 2RW population (Fig. 3).

### Genetic correlations between populations

To assess the genetic similarity of a trait measured in 2 different populations, we calculated the genetic correlation between their marker effects using the multivariate GP model, MP2. In general, the standard error associated with these correlations were quite large, indicating a lot of uncertainty in the estimated genetic correlations between expressions of the same trait in different populations. This was especially the case for the historical data (external population had data until 2020) where datasets were smaller compared to all-years data (external population had data until 2024). The size of the estimated correlations varied a lot depending on trait and which external population was involved. Moderate to high genetic correlations (>0.3) were observed for all traits between 6RW and 2RW, for plant height and rust resistance between 6RW and 6RS, and only for plant height when comparing 6RW with 2RS. For all traits, the genetic correlation was the highest between 6RW and 2RW when considering the all-years data. Interestingly, both the highest and lowest correlations were found for rust resistance, the highest being represented by 6RW and 2RW (0.89 to 0.90) and the lowest by 6RW and 2RS (−0.08 to 0.02) (Table 4).

**Table 4.** Genetic correlations between 6RW and the other populations.

Data	Genetic correlation (*r_g_*) with 6RW		
	Grain yield	Plant height	Rust resistance
Historical			
2RW	0.81 (0.27)	0.65 (0.12)	0.90 (0.16)
6RS	0.43 (1.02E + 05)	0.74 (0.24)	0.38 (0.42)
2RS	0.21 (0.27)	0.71 (0.14)	−0.08 (0.26)
All years			
2RW	0.87 (0.19)	0.84 (0.08)	0.89 (0.12)
6RS	0.23 (0.38)	0.56 (0.24)	0.44 (0.27)
2RS	0.12 (0.27)	0.73 (0.14)	0.02 (0.23)

In parentheses are the standard error of the correlations.

### Impact of additional training years on within-population GP

**Fig. 4.**
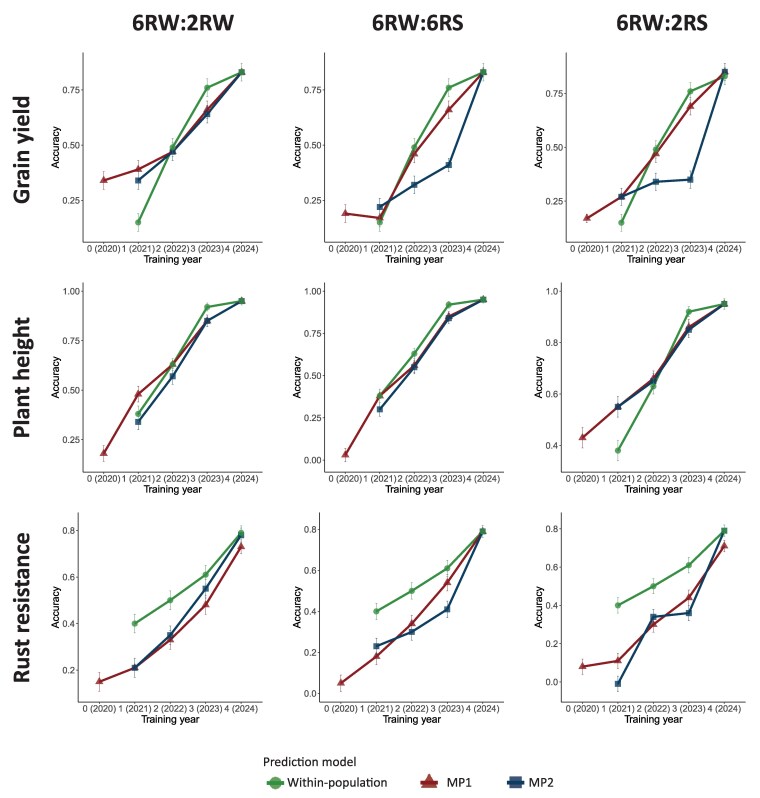
Genomic prediction of 6RW grain yield (top), plant height (middle), and rust resistance (bottom) training on either the 6RW population (green circles) or in combination with historical data from 2RW (left), 6RS (middle), or 2RS (right) using a univariate model (MP1, red triangles) or a multivariate model (MP2, blue squares).

Figure 4 shows the prediction accuracy for grain yield, plant height, and rust resistance in the 6RW population. It compares GP results when the training population consists solely of 6RW individuals (“within-population”) versus a combination of multiple populations (6RW:2RW, 6RW:6RS, or 6RW:2RS). In the latter case, the external training population was accompanied by historical data (observations until year 2020), and 2 different multipopulation models were applied (MP1 and MP2). The models were applied following a CV scheme mimicking the cultivar development process. Here, GP was performed in different training years (i.e. year 0, 1, 2, 3, or 4), which corresponded to the number of years of 6RW data included in the training set. Supplementary Fig. 28 represents a similar analysis, but instead of relying solely on historical data from the external population, it uses all available years of data (all-years data). This was done to investigate whether more data and knowledge of future environments increased prediction accuracy.

For the within-population predictions, accuracy increased as the amount of data increased over years and was maximized when all data were available in training year 4, ending at 0.83 for grain yield, 0.95 for plant height, and 0.79 for rust resistance. Most was to be gained from adding an additional year of data in the early years. Going from training year 1 to training year 2, the prediction accuracy increased from 0.15 to 0.49 for grain yield, from 0.38 to 0.63 for plant height, and from 0.40 to 0.50 for rust resistance. For rust resistance, the increase in prediction accuracy was still substantial when going from training on 3 years of data to 4 years of data. However, for grain yield and plant height, the curve for GP accuracy in 6RW neared a plateau in training year 3, which could indicate that relatively little was to be gained from adding an additional year of data at this point (Fig. 4).

### Performance of the MP1 model versus the within-population model

In training year 0, the training set consisted entirely of data from the external population, as no data were available for the 6RW population. Although the prediction accuracy in this scenario was always lower than when training on at least 2 years of 6RW data, for most traits, it was still possible, with some accuracy, to predict the 6RW phenotypes from a distinct breeding program. The most successful cross-population prediction with an accuracy of 0.42 was observed for plant height when predicted from the extensive 2RS population using either historical data or all-years data. Although initially surprising because of the large genetic differentiation between 2RS and 6RW, we attribute this to the large sample size of 2RS. In addition, grain yield in 6RW was predicted notably better using only 2RW data than using the first year of 6RW alone. On the other hand, some combinations of traits and populations seemed unsuitable for cross-population prediction (accuracy below 0.1), including plant height from 6RS data, rust resistance from 6RS data, and rust resistance from 2RS, but only when using the historical data (Fig. 4, Supplementary Fig. 28). In training year 1, where the 6RW training set was still relatively small (ranging from 49 lines for grain yield to 223 individuals for plant height), both grain yield and plant height showed a gain in accuracy when including the 2RW or the 2RS population in the multipopulation GP model. Once data from all years of 6RW became available, MP1 performed similarly to the within-population model. However, MP1's superior or similar performance to the within-population model was not consistent. Notably, for rust resistance, MP1 performed worse than the within-population model at training years 1 to 3 (Table 1, Fig. 4, Supplementary Fig. 28).

### Performance of MP2 versus MP1

To relax MP1's assumption that a trait measured across populations is genetically correlated with a coefficient of 1, and to allow environmental, spatial, and residual parameters to have different variance estimates across populations, we applied a multivariate GP model, MP2. Surprisingly, we observed no cases of the multivariate MP2 model performing significantly better than the univariate MP1 model, despite our combined datasets covering a wide range of genetic correlations and associated standard errors. On the other hand, we typically observed lower prediction accuracy of MP2 as compared to MP1 and the within-population model in training years 1 to 3. Nevertheless, like MP1, MP2 performed similarly to the within-population model when all years of 6RW data were available (Fig. 4, Supplementary Fig. 28).

### Effect of historical versus all-years data

While using only historical data from an external population simulates the data available for 6RW in training year 0, inclusion of all-years data from the external population increases the size of the training set and may allow multipopulation GP models to gain insight into future environments. Going from multipopulation model training on historical data to all-years data increased the prediction accuracies when the training set was small. This was especially the case for the early years in the breeding program and when the external population was the relatively small 6RS population. The largest increase in accuracy when changing from historical data to all-years data was observed for rust resistance in training year 0 for 2RW, where accuracy changed from 0.15 to 0.35. In year 3, the greatest improvement from using historical data to all-years data (from 0.41 to 0.54) was found for MP2 when including grain yield in 6RS. In contrast, MP1 showed no change and stayed high (0.66). This was not surprising, as the 6RS historical grain yield data included only 1 environment, making it hard to estimate the additional covariance parameters in MP2. When the training set was already very large, i.e. for all traits in combination with 2RS and for grain yield in combination with 2RW, training on all-years data rather than historical data did not change the prediction accuracy of 6RW significantly (Fig. 4, Supplementary Fig. 28).

### Assessing the impact of varying the 6RW training population size

**Fig. 5.**
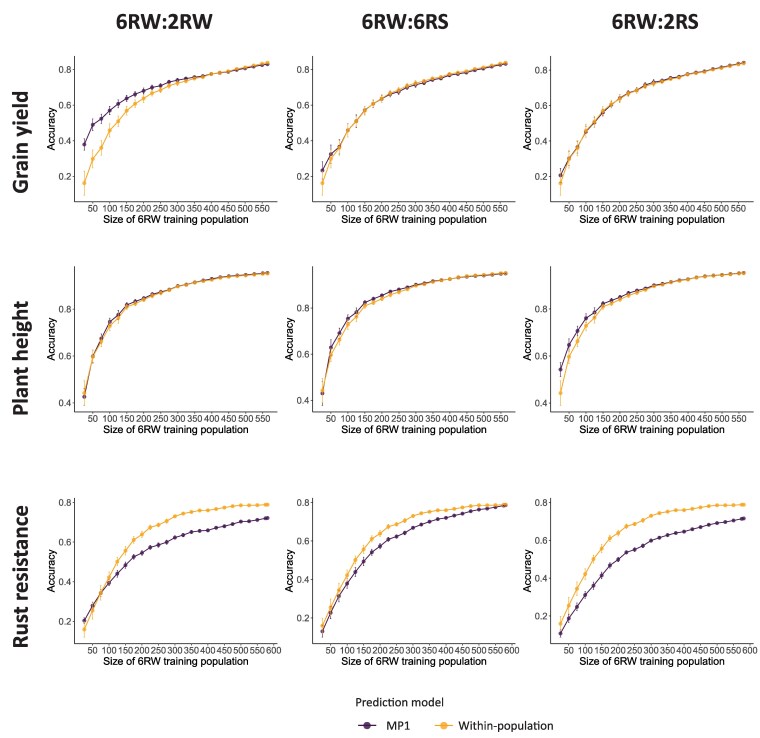
Genomic prediction of 6RW grain yield (upper panel), plant height (middle panel), and rust resistance (lower panel) when the training population consists of the historical 2RW data (left column), 6RS data (middle column), or 2RS data (right column), along with an additional set of randomly selected 6RW individuals added in increments of 25. The results of the within-population model are shown in yellow, while the results of the MP1 model are shown in purple.

To assess how prediction accuracy was affected by increasing sample size while avoiding the confounding effects of age and size of the training population, we applied 2 scenarios: (1) using a randomly subsampled 6RW training population or (2) combining the randomly subsampled 6RW training population with the historical data of an external population using the MP1 model (Fig. 5). Results showed that the prediction accuracy of all traits increased nonlinearly as the 6RW training population grew larger, ultimately coming close to reaching a plateau at the maximum 6RW population size for plant height and rust resistance, while still showing potential to increase for grain yield. Additionally, when sample sizes were similar, a training set of randomly subsampled individuals generally achieved better prediction accuracy than one composed solely of individuals from earlier years. As an example, a set of 225 randomly selected 6RW individuals achieved an average grain yield prediction accuracy of 0.67 (Fig. 5), while the 224 individuals obtained in training year 2 had a prediction accuracy of 0.47 (Fig. 4). This was expected, as the random subsampling procedure increases the extent of close family relations between training and validation sets, while increasing the number of environments represented in the training set (Supplementary Figs. 29 and 30). By comparing the within-population and MP1 models, we found that MP1 had the ability to improve prediction accuracy when the 6RW training size was small enough. However, this only happened for grain yield in combination with the 2RW population and for plant height when combined with 2RS. The former was the more significant of the cases. Here, MP1 performed significantly better than the within-population model, until a large amount of the 6RW lines (*n* > 200) was included in the training population (Fig. 5). In line with what was observed when increasing the 6RW training set over years (Fig. 4), the prediction accuracy of rust resistance typically decreased by combining the 6RW training data with external populations (Fig. 5). Despite the superior performance of MP1 in early training years for plant height in combination with 2RW and grain yield in combination with 2RS (Fig. 4), MP1 did not perform better than the within-population model even for a low number of randomly subsampled 6RW individuals (Fig. 5). This can be attributed to the within-population model's strong performance at small training sizes, which is driven by the greater representation of family and year data in the training in this CV scheme compared to the CV scheme mimicking cultivar development. The former was clearly demonstrated by investigating the relationship between an increasing 6RW training set and the proportion of validation set families represented in the training set. We found that when the training set consisted of only 25 6RW lines, an average of 27% of the validation set families were represented in the training set. The corresponding numbers for training set sizes at 50 and 100 were 50% and 70%, respectively (Supplementary Fig. 29). Similarly, we assessed the average extent of relationships between the training and validation sets at different training set sizes by calculating the average proportion of validation set lines that had at least 1 training set individual with a genetic relationship coefficient greater than 0.5: 72%, 89%, and 97% for training set sizes 25, 50, and 100, respectively (Supplementary Fig. 30). To further highlight the importance of family representation in the training set and to assess whether MP1 still had the ability to improve prediction accuracy when more and more 6RW families were represented in the training set, we repeated the analysis from Fig. 5 with a key modification: instead of selecting 6RW individuals randomly, we maximized family representation. This approach substantially improved prediction accuracy for both models. However, the performance difference between MP1 and the within-population model remained evident, with MP1 continuing to offer an advantage in the same cases as observed in Fig. 5, that is, when the training set was sufficiently small for grain yield in combination with the 2RW population and for plant height when combined with 2RS (Supplementary Fig. 31).

## Discussion

Here, we investigated the circumstances under which the prediction accuracy of grain yield, plant height, and rust resistance in 6RW could be improved by including data from an external breeding population (2RW, 6RS, or 2RS) into the training set. The performance of multipopulation models, univariate (MP1) and multivariate (MP2), and within-population GP models varied for different datasets, sample sizes, traits, and combinations of populations. Our results suggested that including an external population in multipopulation GP benefitted prediction accuracy only when the 6RW training population was small and, in most cases, also biased (only representing few environments) as in the very early stages of new breeding programs. The conclusions for the best choice of model can be summarized into 3 main findings. First, none of the multipopulation models outperformed the within-population model when the genetic correlation of the same trait between populations was close to 0 or when the standard error of the genetic correlation was large. Second, MP2 never resulted in larger prediction accuracy than MP1, even when the theoretical assumptions of MP2 were met, i.e. genetic correlations different from 1, probably because MP2 struggles with estimating its many variance parameters, especially at low sample sizes. Finally, we observed a very similar performance of all 3 prediction approaches when all years of 6RW data were available for training. We attribute this to the fact that, by training year 4, all potential family members of the 6RW individuals to be predicted were already included in the training set, resulting in high genetic relationships between the training and validation sets. For this reason, inclusion of an external population with generally lower genetic relationship coefficients to the validation set offered no benefit. However, in the early training years, many individuals in the validation set would still lack family data or even close relatives in the training set, offering a potential benefit to expanding the training set with individuals from an external, somewhat related population.

### Genetic relationships between training and validation sets

The ability of multipopulation GP models to improve accuracy compared to within-population models depends on many factors. Some of the most critical include the degree of genetic similarity across populations, such as genetic relatedness, shared genetic factors influencing the trait of interest, and reliable estimates of genetic correlations.

GP is most accurate when the training and validation sets are closely related (Wientjes et al. 2013; Lorenz and Smith, 2015). Thus, for prediction accuracy to benefit from multipopulation GP, the combined populations need some degree of genetic similarity. One way to assess genetic similarity between populations is to study the *F*_ST_ values. Previous research has found that prediction accuracy decreases as the *F*_ST_ value between the training and validation sets increases (Scutari et al. 2016). We found that the studied barley subpopulations were highly differentiated, with *F*_ST_ values ranging from 0.24 to 0.38, indicating little exchange of genetic material across breeding programs. In addition to high genetic differentiation between populations, we found that the relationships between 6RW individuals and the 3 other populations were highly concentrated around 0, at first indicating little potential for borrowing information across populations. However, since some cross-population relationships were strong, multipopulation GP still demonstrated potential. In contrast, the genetic relationships within the 6RW population were generally high, largely due to the presence of full-sib data. Consistent with previous studies, we observed that the presence of extended-family data helps substantially in GP, even when working with relatively small sample sizes (Juliana et al. 2019). While this is a major benefit for prediction within a given year, it complicates the practical aspect of breeding, where new crosses are generated each year, and consequently, GP often needs to be applied to unobserved lines that lack full sibs in the training set. However, it is important to note that new lines frequently share at least 1 parent with lines from previous years, which still provides useful genetic relatedness for GP. This is different from the situation of predicting across unrelated populations, as we also observed in this study.

Considering the relationships between the combined populations, it was no surprise that cross-population GP consistently yielded lower prediction accuracy than within-population GP having at least 1 to 2 years of 6RW training data available. Similar results have been observed in several plant species, e.g. maize (Technow et al. 2013), potato (Sverrisdóttir et al. 2018), wheat (Michel et al. 2021), and apple (Cazenave et al. 2022). Although the worse performance of cross-population GP compared to within-population GP remains a challenge in the practical aspects of breeding, our results reveal that, even in the absence of full-sib, half-sib, or parent data, it is often still possible to do cross-population GP and get a better prediction accuracy than expected by random. Thus, our findings highlight the promise of cross-population prediction, not only as a valuable tool for newly established breeding programs but also as a powerful strategy for well-established breeding programs, where unobserved traits, previously assessed in other populations, need to be predicted.

Because prediction accuracy depends strongly on the genetic relatedness between training and validation sets, identifying the most informative individuals to include in the training set can be critical, especially when phenotyping resources are limited. While our multipopulation prediction approach used all available data from the 6RW population in a given training year, combined with data from the external population (either historical or all years), future work could explore training set optimization to select the optimal set of related individuals to maximize prediction accuracy.

### Genetic correlations between populations

An additional *true* parameter that would need to be significantly different from 0 for multipopulation GP to potentially outperform the within-population model is the genetic correlation of the same trait when measured in different populations. Like Lehermeier et al. (2015), we found that genetic correlations between populations tended to be higher when populations were genetically more similar and, conversely, lower when populations were more genetically differentiated. However, we also observed cases where the genomic distance was large, yet genetic correlations for certain traits were still high.

While genetic correlations can vary between traits and populations, the larger the genetic correlation, the larger the potential benefit of combining populations for GP (Porto-Neto et al. 2015). In line with this, we observed that multipopulation GP did not outperform the within-population model when genetic correlations were low, as observed for rust resistance when using 2RS as the external population. We speculate that the low genetic correlation of rust resistance in 6RW and 2RS could be due to different resistance genes, different growth habits leaving different times for fungi to attack, or a combination of both. Supporting this hypothesis, recent studies have found that growth-type (winter or spring) specific QTLs of leaf rust do exist (Kunze et al. 2024).

Despite the estimated genetic correlation of grain yield in 6RW and 2RS not differing from 0 when considering standard errors, the multipopulation GP models still outperformed the within-population model in training year 1. At the present, we have no clear explanation for this.

### The challenge of parameter estimations from small datasets

While MP1 assumes a genetic correlation of 1 between the same trait measured in different populations, which may introduce potential biases, MP2 attempts to estimate these correlations, albeit with possible estimation errors. For this reason, another factor to consider for potential success of multipopulation GP is the standard errors of the genetic correlations. Large standard errors can arise from small sample sizes and/or large trait variation across populations. The latter helps explain why the correlations between 6RW and the spring types (6RS and 2RS) typically were accompanied by the largest standard errors. In addition, the small 6RS sample size results in the combination of 6RW and 6RS being unfavorable across all traits. However, what remains puzzling is that 2RW failed to increase prediction accuracy of rust resistance in 6RW, despite the genetic correlation being high with relatively low standard error. One possible explanation could be that a few markers linked to large effect loci are shared between the populations, inflating the genetic correlation without contributing to prediction accuracy.

Though initially surprising, we never observed instances of MP2 performing significantly better than the other models, despite the estimated genetic correlations favoring MP2 by being significantly different from 1. The estimated genetic correlations of plant height between 6RW and 2RW as well as between 6RW and 2RS were moderate to high while differing from 1. Still, we observed that MP1 achieved better prediction accuracy in training year 1 than MP2. We believed that the reason for this, and the general struggle of MP2 to outperform MP1, was due to MP2 requiring significantly more data for parameter estimation than MP1. Specifically, MP2 estimates more than twice the numbers of parameters than MP1, resulting in much less precise estimations, particularly in training years where the dataset is small.

One of the most comprehensive studies of multipopulation GP in plants to date was conducted by Lehermeier et al. (2015). Similar to our studies, the authors applied 2 multipopulation models (univariate and multivariate) as well as a single-population GP model to several plant populations in rice, maize, and wheat. Consistent with our findings, Lehermeier et al. (2015) found that multipopulation GP models had the ability to outperform the within-population GP model only when the combined populations were genetically related, with nonzero genetic correlation for traits measured across populations. This was most evident in their studied maize and wheat populations. In maize, where the within-subpopulation sample sizes were small, the researchers found that the univariate multipopulation model performed better than the multivariate multipopulation model. In wheat, where subpopulation sizes were larger, the multivariate multipopulation model offered similar performance to or marginally better performance than the univariate multipopulation model. These effects of genetic heterogeneity and sample size on GP accuracy align well with our findings. Interestingly, the lack of improvement in the multivariate multipopulation (MP2) model over the simple univariate multipopulation (MP1) model has also been observed in apple, despite moderate and precisely estimated genetic correlations and large sample sizes (Cazenave et al. 2022).

### The focal population size and its implications

Another factor that affects the ability of multipopulation GP to outperform within-population GP is the size of the focal population. Although it might seem surprising, our results suggest that multipopulation GP has the potential to increase GP accuracy only in the early years of a breeding program. In line with our findings, previous studies have shown that performing multipopulation GP can increase the prediction accuracy when the training set is small enough. Technow et al. (2013) found that combining 2 small distinct subpopulations of maize (dent and flint) in a training population of maximum 150 accessions increased the overall prediction ability for both groups. Due to the small size of each subpopulation, the researchers were unable to test if the combined approach would still be beneficial if the focal population was larger. Further, Technow and Totir (2015) used computer simulations to conclude that there is generally little benefit in including an external population in GP if the focal population is already sufficiently large, i.e. consisting of at least 50 individuals in their study. A similar pattern was observed in winter wheat, where researchers found that multipopulation GP could be helpful if the focal population was small (*n* = 87), but it only led to marginal improvements in prediction accuracy when the focal population was relatively large (*n* = 611) (Michel et al. 2021). At larger samples sizes, other studies have found that expanding a barley training population with external populations might have no effect on GP accuracy or even reduce it. Lorenz and Smith (2015) found that by adding increasingly unrelated individuals to a 6-rowed barley training population, the prediction accuracy for plant height, deoxynivalenol concentrations, and resistance to *Fusarium* head blight in 6-rowed barley decreased. However, the authors had very low SNP density (342 SNPs), complicating the joint analyses of different populations, since the linkage between markers and causal genes most likely was not be conserved. In addition, Lorenz and Smith (2015) applied only univariate multipopulation models. Another example is provided by Ankamah-Yeboah et al. (2020), who combined two 2RW barley breeding programs from different Danish breeding companies. In their study, the combined training set either decreased or did not alter the overall predictive ability of the focal population. However, like Lorenz and Smith (2015), the authors exclusively applied univariate models and relied on relatively few SNP markers (4830 SNPs) compared to our study.

### Comparison of historical and all-years data

To investigate whether including years from the validation set into the training set improves the model performance, we compared 2 validation scenarios: one where the training set included only data from years prior to the validation set, ensuring no year overlap between sets (historical data, until 2020), and another where the training set included both past years and years from the validation set (all-years data, until 2024). The similar performance of the multipopulation model on historical and all-years data in populations with extensive data suggests that the advantage of all-years data is not due to overlapping environments between populations, i.e. shared environmental effects, but rather the large volume of data in the external populations. On the other hand, we observed that expanding the 6RW training set with additional years of data often improved the prediction accuracy substantially. While this may suggest the importance of having a training set that is representative of the years included in the validation set, we must consider that, in our dataset, family data and training years are highly correlated. Therefore, we cannot say with certainty that the key factor is not the inclusion of highly related family data in the training set, as it may very well be a significant contributor to the observed improvements.

Consistent with our findings, Jarquin et al. (2016) also found that large datasets can overcome the lack of shared GxE effects between the training and validation sets, especially when environments are considered as individual fields rather than geographical locations across years. Further, we should emphasize that the number of shared environments between our studied populations is small, as 6RW and the spring populations have distinct growing seasons. In addition, our multipopulation models do not take any advantage of some fields being shared between populations, as environments were nested within populations. This can explain the finding that all-years data from 2RW did not improve 6RW grain yield prediction accuracy, despite similar locations (but distinct fields) and the large sample size in 2RW. While such analyses are beyond the scope of the current study, future research could focus on modeling environmental similarities between populations to better disentangle genomic breeding values from environment-specific breeding values. This could be done by predicting line performance within environments, rather than across multiple environments. Another potential direction is to dynamically adjust the size of the external training population in each training year to better reflect realistic data availability. In this study, we opted to use a fixed external training set to ensure comparability across training years. However, adapting the external dataset over time may further enhance prediction accuracy in practical breeding applications.

### Improving genomic models of population heterogeneity

In some cattle studies, attempts to improve GP accuracy by adding an external population in a multivariate multipopulation GP model appear to have been more successful, likely due to more genetic recombination between subpopulations and/or reduced GxE within and between them. Previous research has shown that expanding the training set with geographically distinct populations using bivariate or trivariate multipopulation models can consistently improve prediction accuracy (De Haas et al. 2012). Other studies in livestock have found that while combining different purebred populations does not inherently increase GP accuracy, incorporating population mixtures into the training set helps connect purebred groups and increase accuracy (Toosi et al. 2010; Karaman et al. 2021). While creating population mixtures of barley is one promising strategy to enhance multipopulation GP, it can be costly and resource intensive. Alternatively, improvement in GP accuracy may be achieved by relaxing certain model assumptions. For example, standard Genomic BLUP models assume that marker effects follow the same distribution; however, one way to relax this assumption could be to categorize SNPs into different groups based on certain characteristics and model their effects separately. Previous research suggests that extending the multivariate multipopulation GP model to include 2 **G** matrices—one built from SNPs with prior knowledge and the other from remaining SNPs—can significantly boost prediction accuracy compared to models with only 1 **G** matrix and single-population models with 2 corresponding matrices (Raymond et al. 2018; Raymond et al. 2020). However, it should be stated that this approach depends on having accurate prior information about SNPs with substantial effects on the trait of interest.

An alternative way to categorize SNPs and address a potential limitation of the applied MP2 model—the assumption that genetic correlations between populations are consistent across the entire genome—was proposed by Chen et al. (2014). By allowing heterogeneous covariance of SNP effects between populations, the authors introduced a more refined approach to better integrate different populations in multipopulation GP.

Finally, previous research on the 4 barley populations studied here has shown that some degree of population admixture exists within populations (Skovbjerg et al. 2025). While population-specific SNP effects are accounted for in our applied MP2 model, population memberships are still modeled as discrete variables. Rio et al. (2020) demonstrated that modeling population membership as a nondiscrete variable by incorporating admixture coefficients can improve prediction accuracy when combining populations. This strategy may offer a useful approach for improving our future studies on multipopulation GP.

### Accounting for environmental heterogeneity in multipopulation models

In this study, we modeled phenotypic covariance between environments through general genomic values (**g**_a_ and **g**_l_) and accounted for GxE through environment-specific genomic values (**w** and **v**). However, we did not model variance heterogeneity or correlations of GxE effects across environments. Capturing such heterogeneity and correlations requires complex models that either treat environments as separate response variables or estimate an unstructured covariance among those. These models are complex (particularly when population heterogeneity is also modeled, as in MP2), computationally demanding (especially with REML-based optimizers such as those in DMU), and unable to predict outcomes in new environments (e.g. in locations where a given population has not been tested). Advanced GxE modeling may improve the accuracy of population-specific genetic effects, but applying it to our multipopulation, multienvironment data would require several adjustments: (1) reducing the dimensionality of data across many environments, for example with factor-analytic methods (Runcie et al. 2021); (2) adopting more efficient solvers for fitting complex models, such as pseudo-expectation Gauss–Seidel algorithms (Xavier and Habier 2022; Xavier et al. 2025); and (3) extending models to incorporate environmental covariates, for example with extended MegaLMM (Hu et al. 2025). Comprehensive modeling of both population and environmental heterogeneity could offer a fully flexible framework for GP, but its practical value in plant breeding—particularly regarding prediction accuracy—remains to be established.

## Supplementary Material

jkaf218_Supplementary_Data

## Data Availability

The data underlying this article are available in Supplementary Tables 4 to 8. Genotype data can be accessed at https://doi.org/10.6084/m9.figshare.28748273.v1. Supplemental material available at G3 online.
