# Supplementary material for: Genomic prediction in a small barley population can benefit from training on related populations
Source: G3 (Bethesda). 2025 Oct 23;15(11):jkaf218. doi: 10.1093/g3journal/jkaf218 (PMC12610402; doi:10.1093/g3journal/jkaf218)
Supplement: jkaf218_Supplementary_Data [file jkaf218_supplementary_data.zip › Figure_S28_G3-2025-406199.pdf]

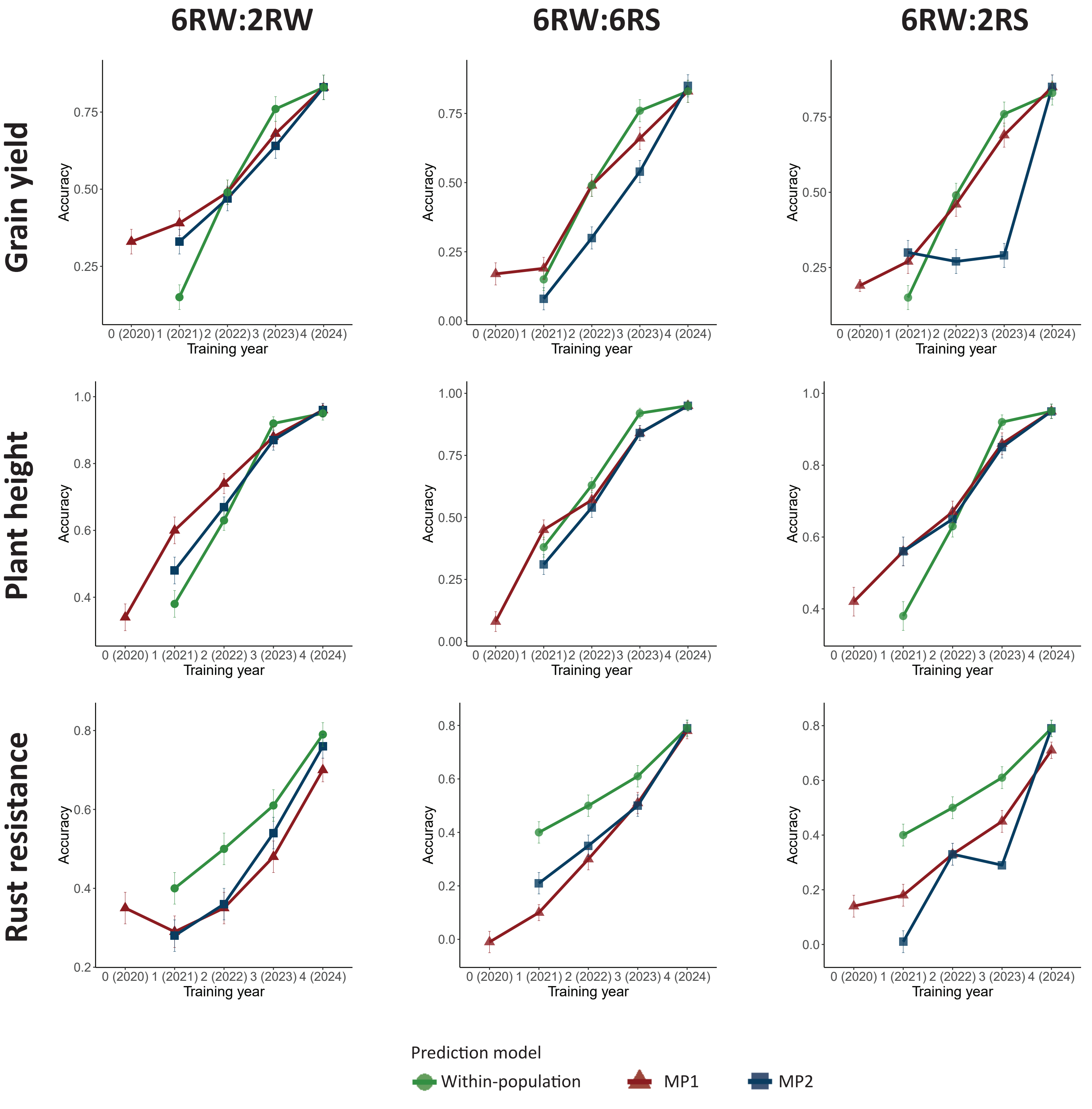
6RW:2RW
6RW:6RS
6RW:2RS
Grain yield
Plant height
Rust resistance
Accuracy
Training year
0 (2020)
1 (2021)
2 (2022)
3 (2023)
4 (2024)
Prediction model
Within-population
MP1
MP2
