# Supplementary material for: Genomic prediction in a small barley population can benefit from training on related populations
Source: G3 (Bethesda). 2025 Oct 23;15(11):jkaf218. doi: 10.1093/g3journal/jkaf218 (PMC12610402; doi:10.1093/g3journal/jkaf218)
Supplement: jkaf218_Supplementary_Data [file jkaf218_supplementary_data.zip › Figure_S4_G3-2025-406199.pdf]

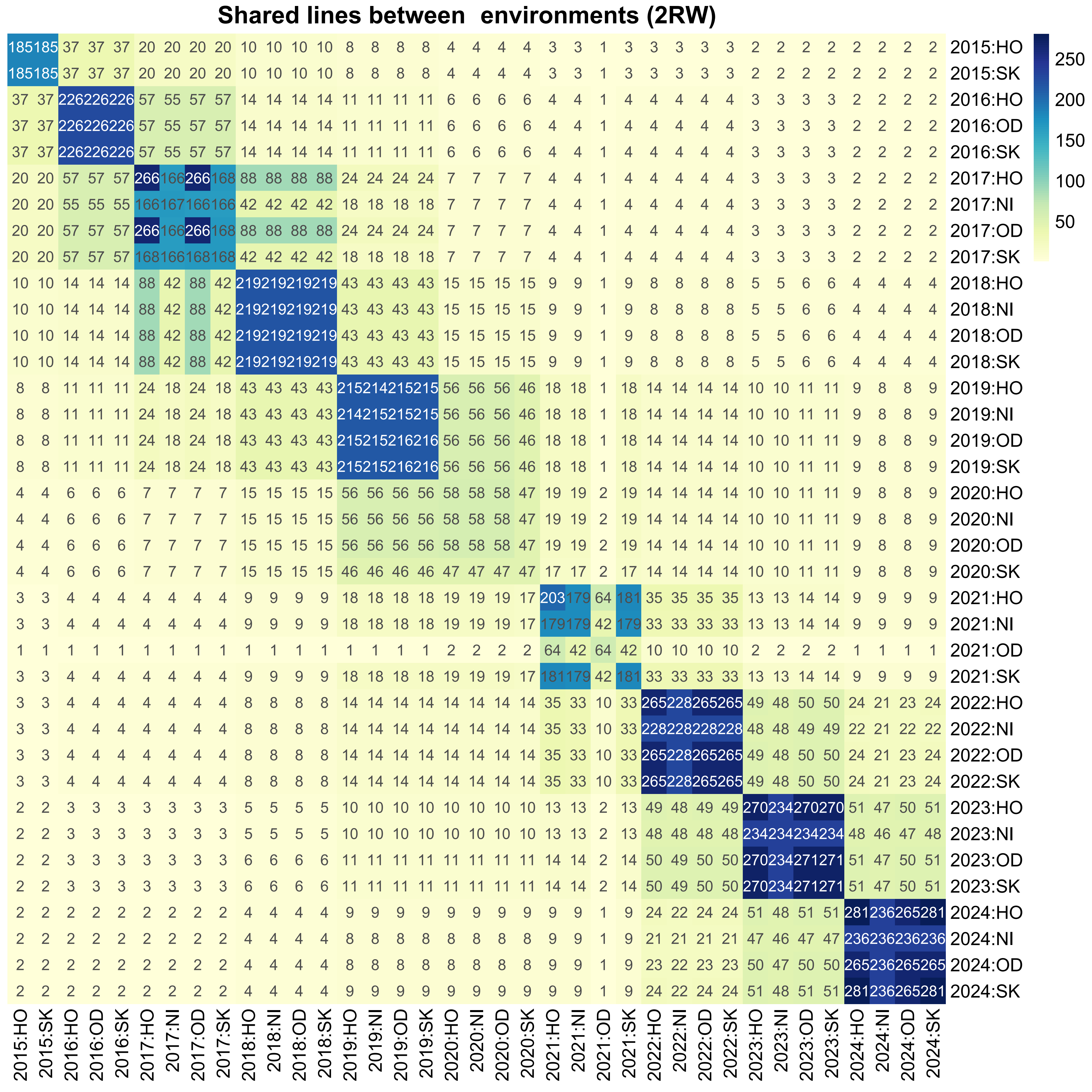

Shared lines between environments (2RW)
185 185 37 37 37 20 20 20 20 10 10 10 10 8 8 8 8 4 4 4 4 3 3 1 3 3 3 3 3 2 2 2 2 2 2 2 2 2015:HO
185 185 37 37 37 20 20 20 20 10 10 10 10 8 8 8 8 4 4 4 4 3 3 1 3 3 3 3 3 2 2 2 2 2 2 2 2 2015:SK
37 37 226 226 226 57 55 57 57 14 14 14 14 11 11 11 11 6 6 6 6 4 4 1 4 4 4 4 4 3 3 3 3 2 2 2 2 2016:HO
37 37 226 226 226 57 55 57 57 14 14 14 14 11 11 11 11 6 6 6 6 4 4 1 4 4 4 4 4 3 3 3 3 2 2 2 2 2016:OD
37 37 226 226 226 57 55 57 57 14 14 14 14 11 11 11 11 6 6 6 6 4 4 1 4 4 4 4 4 3 3 3 3 2 2 2 2 2016:SK
20 20 57 57 57 266 166 266 168 88 88 88 88 24 24 24 24 7 7 7 7 4 4 1 4 4 4 4 4 3 3 3 3 2 2 2 2 2017:HO
20 20 55 55 55 166 167 166 166 42 42 42 42 18 18 18 18 7 7 7 7 4 4 1 4 4 4 4 4 3 3 3 3 2 2 2 2 2017:NI
20 20 57 57 57 266 166 266 168 88 88 88 88 24 24 24 24 7 7 7 7 4 4 1 4 4 4 4 4 3 3 3 3 2 2 2 2 2017:OD
20 20 57 57 57 168 166 168 168 42 42 42 42 18 18 18 18 7 7 7 7 4 4 1 4 4 4 4 4 3 3 3 3 2 2 2 2 2017:SK
10 10 14 14 14 88 42 88 42 219 219 219 219 43 43 43 43 15 15 15 15 9 9 1 9 8 8 8 8 5 5 6 6 4 4 4 4 2018:HO
10 10 14 14 14 88 42 88 42 219 219 219 219 43 43 43 43 15 15 15 15 9 9 1 9 8 8 8 8 5 5 6 6 4 4 4 4 2018:NI
10 10 14 14 14 88 42 88 42 219 219 219 219 43 43 43 43 15 15 15 15 9 9 1 9 8 8 8 8 5 5 6 6 4 4 4 4 2018:OD
10 10 14 14 14 88 42 88 42 219 219 219 219 43 43 43 43 15 15 15 15 9 9 1 9 8 8 8 8 5 5 6 6 4 4 4 4 2018:SK
8 8 11 11 11 24 18 24 18 43 43 43 43 215 214 215 215 56 56 56 46 18 18 1 18 14 14 14 14 10 10 11 11 9 8 8 9 2019:HO
8 8 11 11 11 24 18 24 18 43 43 43 43 214 215 215 215 56 56 56 46 18 18 1 18 14 14 14 14 10 10 11 11 9 8 8 9 2019:NI
8 8 11 11 11 24 18 24 18 43 43 43 43 215 215 216 216 56 56 56 46 18 18 1 18 14 14 14 14 10 10 11 11 9 8 8 9 2019:OD
8 8 11 11 11 24 18 24 18 43 43 43 43 215 215 216 216 56 56 56 46 18 18 1 18 14 14 14 14 10 10 11 11 9 8 8 9 2019:SK
4 4 6 6 6 7 7 7 7 15 15 15 15 56 56 56 56 58 58 58 47 19 19 2 19 14 14 14 14 10 10 11 11 9 8 8 9 2020:HO
4 4 6 6 6 7 7 7 7 15 15 15 15 56 56 56 56 58 58 58 47 19 19 2 19 14 14 14 14 10 10 11 11 9 8 8 9 2020:NI
4 4 6 6 6 7 7 7 7 15 15 15 15 56 56 56 56 58 58 58 47 19 19 2 19 14 14 14 14 10 10 11 11 9 8 8 9 2020:OD
4 4 6 6 6 7 7 7 7 15 15 15 15 46 46 46 46 47 47 47 47 17 17 2 17 14 14 14 14 10 10 11 11 9 8 8 9 2020:SK
3 3 4 4 4 4 4 4 4 9 9 9 9 18 18 18 18 19 19 19 17 203 179 64 181 35 35 35 35 13 13 14 14 9 9 9 9 2021:HO
3 3 4 4 4 4 4 4 4 9 9 9 9 18 18 18 18 19 19 19 17 179 179 42 179 33 33 33 33 13 13 14 14 9 9 9 9 2021:NI
1 1 1 1 1 1 1 1 1 1 1 1 1 1 1 1 1 2 2 2 2 64 42 64 42 10 10 10 10 2 2 2 2 1 1 1 1 2021:OD
3 3 4 4 4 4 4 4 4 9 9 9 9 18 18 18 18 19 19 19 17 181 179 42 181 33 33 33 33 13 13 14 14 9 9 9 9 2021:SK
3 3 4 4 4 4 4 4 4 8 8 8 8 14 14 14 14 14 14 14 14 35 33 10 33 265 228 265 265 49 48 50 50 24 21 23 24 2022:HO
3 3 4 4 4 4 4 4 4 8 8 8 8 14 14 14 14 14 14 14 14 35 33 10 33 228 228 228 228 48 48 49 49 22 21 22 22 2022:NI
3 3 4 4 4 4 4 4 4 8 8 8 8 14 14 14 14 14 14 14 14 35 33 10 33 265 228 265 265 49 48 50 50 24 21 23 24 2022:OD
3 3 4 4 4 4 4 4 4 8 8 8 8 14 14 14 14 14 14 14 14 35 33 10 33 265 228 265 265 49 48 50 50 24 21 23 24 2022:SK
2 2 3 3 3 3 3 3 3 5 5 5 5 10 10 10 10 10 10 10 10 13 13 2 13 49 48 49 49 270 234 270 270 51 47 50 51 2023:HO
2 2 3 3 3 3 3 3 3 5 5 5 5 10 10 10 10 10 10 10 10 13 13 2 13 48 48 48 48 234 234 234 234 48 46 47 48 2023:NI
2 2 3 3 3 3 3 3 3 6 6 6 6 11 11 11 11 11 11 11 11 14 14 2 14 50 49 50 50 270 234 271 271 51 47 50 51 2023:OD
2 2 3 3 3 3 3 3 3 6 6 6 6 11 11 11 11 11 11 11 11 14 14 2 14 50 49 50 50 270 234 271 271 51 47 50 51 2023:SK
2 2 2 2 2 2 2 2 2 4 4 4 4 9 9 9 9 9 9 9 9 9 9 1 9 24 22 24 24 51 48 51 51 281 236 265 281 2024:HO
2 2 2 2 2 2 2 2 2 4 4 4 4 8 8 8 8 8 8 8 8 9 9 1 9 21 21 21 21 47 46 47 47 236 236 236 236 2024:NI
2 2 2 2 2 2 2 2 2 4 4 4 4 8 8 8 8 8 8 8 8 9 9 1 9 23 22 23 23 50 47 50 50 265 236 265 265 2024:OD
2 2 2 2 2 2 2 2 2 4 4 4 4 9 9 9 9 9 9 9 9 9 9 1 9 24 22 24 24 51 48 51 51 281 236 265 281 2024:SK
2015:HO 2015:SK 2016:HO 2016:OD 2016:SK 2017:HO 2017:NI 2017:OD 2017:SK 2018:HO 2018:NI 2018:OD 2018:SK 2019:HO 2019:NI 2019:OD 2019:SK 2020:HO 2020:NI 2020:OD 2020:SK 2021:HO 2021:NI 2021:OD 2021:SK 2022:HO 2022:NI 2022:OD 2022:SK 2023:HO 2023:NI 2023:OD 2023:SK 2024:HO 2024:NI 2024:OD 2024:SK
250
200
150
100
50
