# Supplementary material for: Genomic prediction in a small barley population can benefit from training on related populations
Source: G3 (Bethesda). 2025 Oct 23;15(11):jkaf218. doi: 10.1093/g3journal/jkaf218 (PMC12610402; doi:10.1093/g3journal/jkaf218)
Supplement: jkaf218_Supplementary_Data [file jkaf218_supplementary_data.zip › Supplemental_Material_Legends_G3-2025-406199.docx]

**Description of supplementary files**

**Figure S1.** Flow chart of quality control steps.

**Figure S2.** Histograms showing the number of SNP allele mismatches between all pairs of individuals in the 6RW, 2RW, 6RS, or the 2RS population. The *x*-axis shows the number allelic differences between two individuals based on 13,035 SNPs (26,070 alleles in total). The *y*-axis indicates how many such pairs were observed for each mismatch count. Red vertical lines indicate the genetic redundancy threshold of 100 SNP alleles. The blue lines display the population-specific average number of allelic differences between two individuals; the exact value is shown above the blue line.

**Figure S3.** Heatmap showing the number of lines shared between pairs of environments within 6RW for grain yield. Each cell displays the count of lines evaluated in both environments, with darker shades representing higher overlap. Diagonal cells display the total number of lines evaluated in each environment.

**Figure S4.** Heatmap showing the number of lines shared between pairs of environments within 2RW for grain yield. Each cell displays the count of lines evaluated in both environments, with darker shades representing higher overlap. Diagonal cells display the total number of lines evaluated in each environment.

**Figure S5.** Heatmap showing the number of lines shared between pairs of environments within 6RS for grain yield. Each cell displays the count of lines evaluated in both environments, with darker shades representing higher overlap. Diagonal cells display the total number of lines evaluated in each environment.

**Figure S6.** Heatmap showing the number of lines shared between pairs of environments within 2RS for grain yield. Each cell displays the count of lines evaluated in both environments, with darker shades representing higher overlap. Diagonal cells display the total number of lines evaluated in each environment.

**Figure S7.** Heatmap showing the number of lines shared between pairs of environments within 6RW for plant height. Each cell displays the count of lines evaluated in both environments, with darker shades representing higher overlap. Diagonal cells display the total number of lines evaluated in each environment.

**Figure S8.** Heatmap showing the number of lines shared between pairs of environments within 2RW for plant height. Each cell displays the count of lines evaluated in both environments, with darker shades representing higher overlap. Diagonal cells display the total number of lines evaluated in each environment.

**Figure S9.** Heatmap showing the number of lines shared between pairs of environments within 6RS for plant height. Each cell displays the count of lines evaluated in both environments, with darker shades representing higher overlap. Diagonal cells display the total number of lines evaluated in each environment.

**Figure S10.** Heatmap showing the number of lines shared between pairs of environments within 2RS for plant height. Each cell displays the count of lines evaluated in both environments, with darker shades representing higher overlap. Diagonal cells display the total number of lines evaluated in each environment.

**Figure S11.** Heatmap showing the number of lines shared between pairs of environments within 6RW for rust resistance. Each cell displays the count of lines evaluated in both environments, with darker shades representing higher overlap. Diagonal cells display the total number of lines evaluated in each environment.

**Figure S12.** Heatmap showing the number of lines shared between pairs of environments within 2RW for rust resistance. Each cell displays the count of lines evaluated in both environments, with darker shades representing higher overlap. Diagonal cells display the total number of lines evaluated in each environment.

**Figure S13.** Heatmap showing the number of lines shared between pairs of environments within 6RS for rust resistance. Each cell displays the count of lines evaluated in both environments, with darker shades representing higher overlap. Diagonal cells display the total number of lines evaluated in each environment.

**Figure S14.** Heatmap showing the number of lines shared between pairs of environments within 2RS for rust resistance. Each cell displays the count of lines evaluated in both environments, with darker shades representing higher overlap. Diagonal cells display the total number of lines evaluated in each environment.

**Figure S15.** PCA plot showing PC1 versus PC3. Points (individuals) are coloured and shaped according to the population to which they belong.

**Figure S16**. Histograms of grain yield observations per year and location in the 6RW. The red dashed lines indicate the median in a given year x location combination.

**Figure S17**. Histograms of grain yield observations per year and location in the 2RW. The red dashed lines indicate the median in a given year x location combination.

**Figure S18**. Histograms of grain yield observations per year and location in the 6RS. The red dashed lines indicate the median in a given year x location combination.

**Figure S19**. Histograms of grain yield observations per year and location in the 2RS. The red dashed lines indicate the median in a given year x location combination.

**Figure S20**. Histograms of plant height observations per year and location in the 6RW. The red dashed lines indicate the median in a given year x location combination.

**Figure S21**. Histograms of plant height observations per year and location in the 2RW. The red dashed lines indicate the median in a given year x location combination.

**Figure S22**. Histograms of plant height observations per year and location in the 6RS. The red dashed lines indicate the median in a given year x location combination.

**Figure S23**. Histograms of plant height observations per year and location in the 2RS. The red dashed lines indicate the median in a given year x location combination.

**Figure S24**. Histograms of rust resistance observations per year and location in the 6RW. The red dashed lines indicate the median in a given year x location combination.

**Figure S25**. Histograms of rust resistance observations per year and location in the 2RW. The red dashed lines indicate the median in a given year x location combination.

**Figure S26**. Histograms of rust resistance observations per year and location in the 6RS. The red dashed lines indicate the median in a given year x location combination.

**Figure S27**. Histograms of rust resistance observations per year and location in the 2RS. The red dashed lines indicate the median in a given year x location combination.

**Figure S28.** Genomic prediction of 6RW grain yield (top), plant height (middle) and rust resistance (bottom) training on either the 6RW population (green circles), or in combination with all-years data 2RW (left), 6RS (middle) or 2RS (right) using a univariate model (MP1, red triangles) or a multivariate model (MP2, blue squares).

**Figure S29.** Relationship between the 6RW training population size and proportion of non-training 6RW individuals with represented families. The graph illustrates how different sizes of a 6RW training population (*x*-axis) correspond to a proportion of remaining non-training 6RW individuals that have full sibs in the training population (*y*-axis). Each point represents the average of 10,000 random samplings.

**Figure S30.** Relationship between the 6RW training population size and proportion of non-training 6RW individuals with close relatives. The graph illustrates how different sizes of a 6RW training population (*x*-axis) correspond to a proportion of remaining non-training 6RW individuals that have close relatives (genomic relationship coefficient above 0.5) in the training population (*y*-axis). Each point represents the average of 10,000 random samplings.

**Figure S31.** Genomic prediction of 6RW grain yield (upper panel), plant height (middle panel), and rust resistance (lower panel) when the training population consists of the historical 2RW data (left column), 6RS data (middle column) or 2RS data (right column), along with an additional set of selected 6RW individuals added in increments of 25. These selected 6RW individuals are chosen to represent the largest possible number of families in the 6RW population. The results of the within-population model are shown in pink, while the results of the MP1 model are shown in green.

**Table S1.** Overview of the locations with phenotypic observations of different traits by year and population. Numbers denote the number of phenotypic observations in a given combination of year and location.

**Table S2.** The scoring scale for leaf rust resistance.

**Table S3.** The distribution of cross-population relationships.

**Table S4.** Grain yield phenotypes used for genomic predictions.

**Table S5.** Plant height phenotypes used for genomic predictions.

**Table S6.** Rust resistance phenotypes used for genomic predictions.

**Table S7.** List of SNP markers that passed quality control and was used for genomic prediction.
