# Supplementary figures and images for: Genomic prediction in a small barley population can benefit from training on related populations
Source: G3 (Bethesda). 2025 Oct 23;15(11):jkaf218. doi: 10.1093/g3journal/jkaf218 (PMC12610402; doi:10.1093/g3journal/jkaf218)

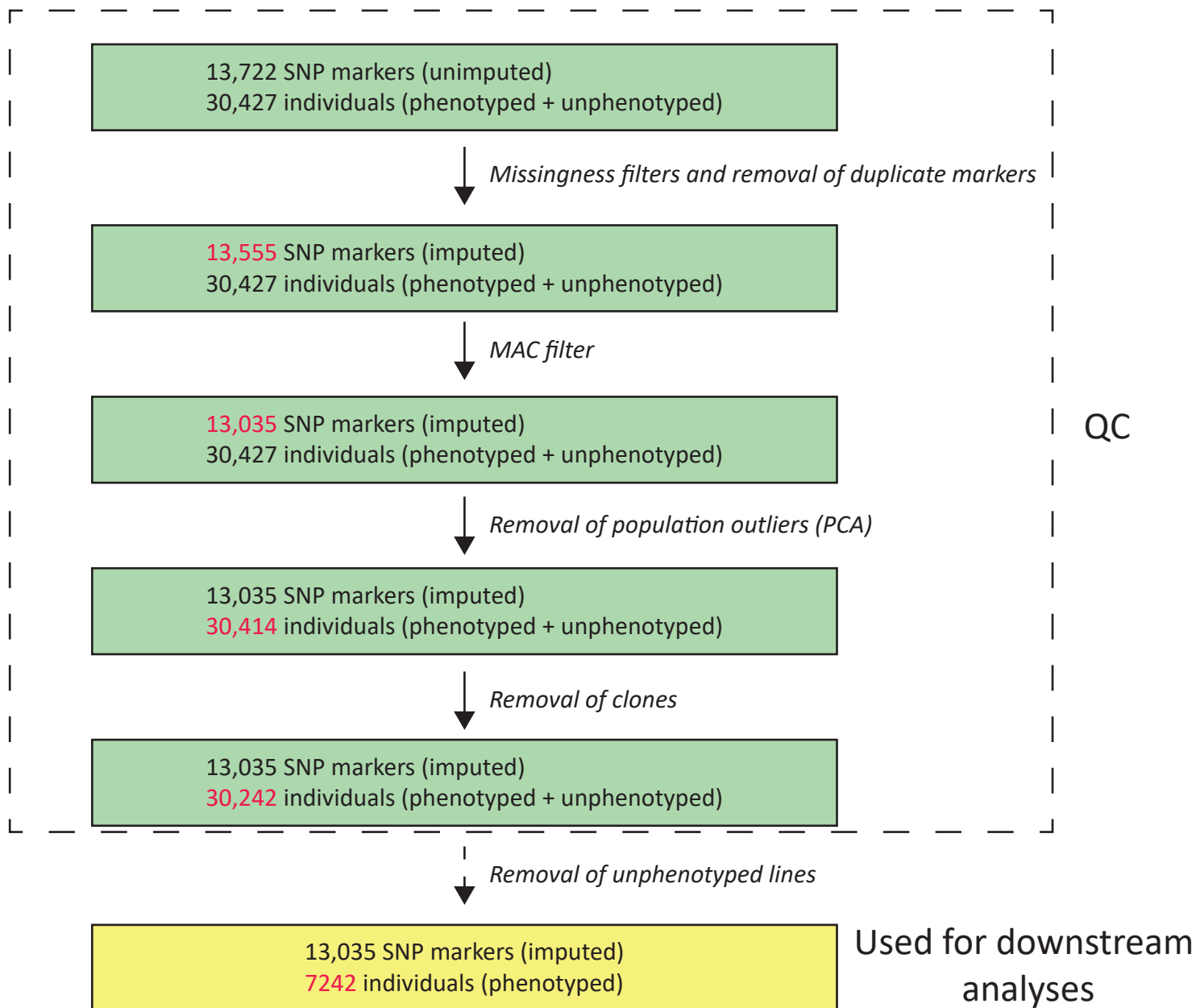

Supplement: jkaf218_Supplementary_Data [file jkaf218_supplementary_data.zip › Figure_S1_G3-2025-406199.pdf]

Shared lines between environments (2RS)

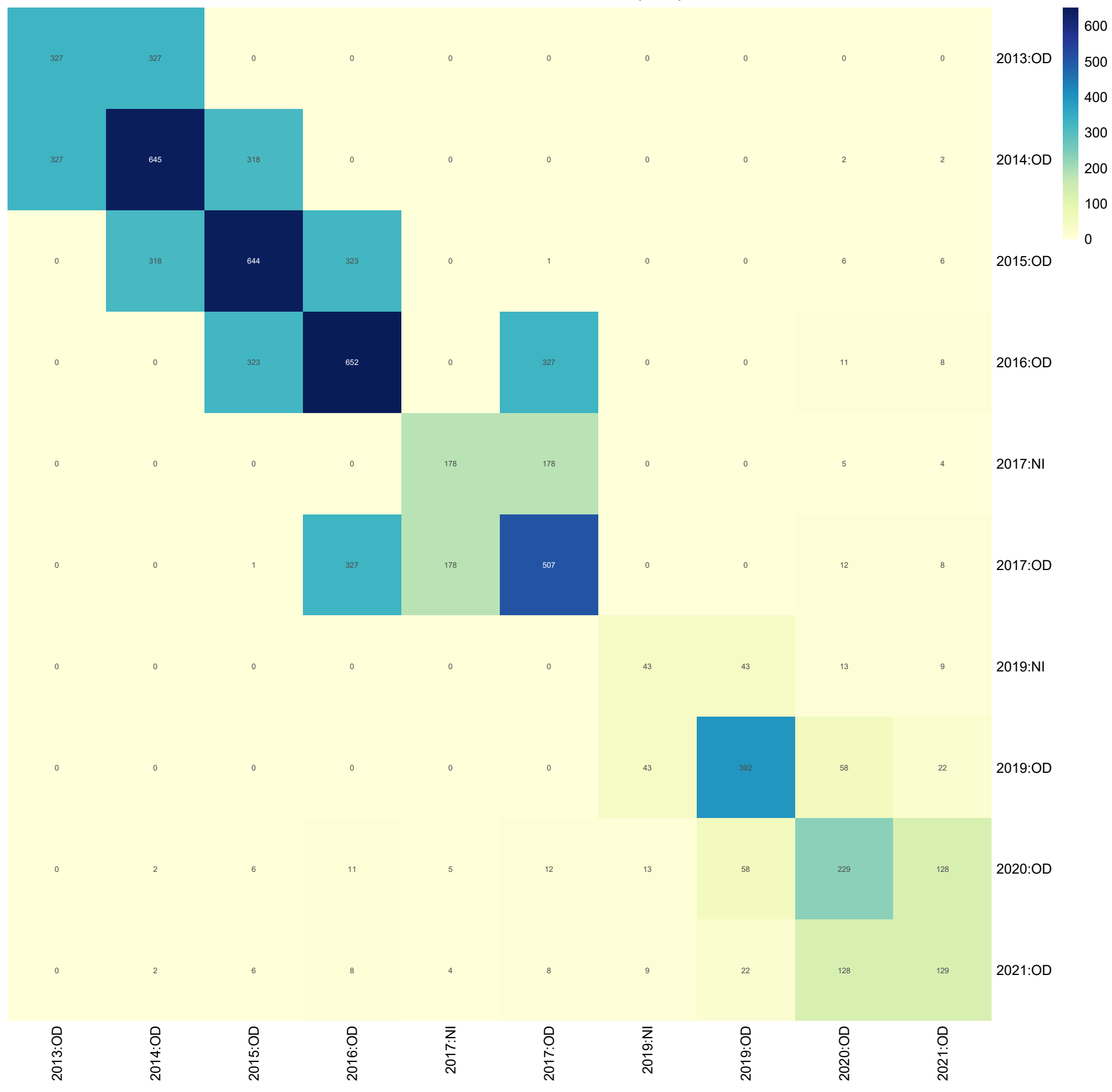

Supplement: jkaf218_Supplementary_Data [file jkaf218_supplementary_data.zip › Figure_S10_G3-2025-406199.pdf]

Shared lines between environments (6RW)

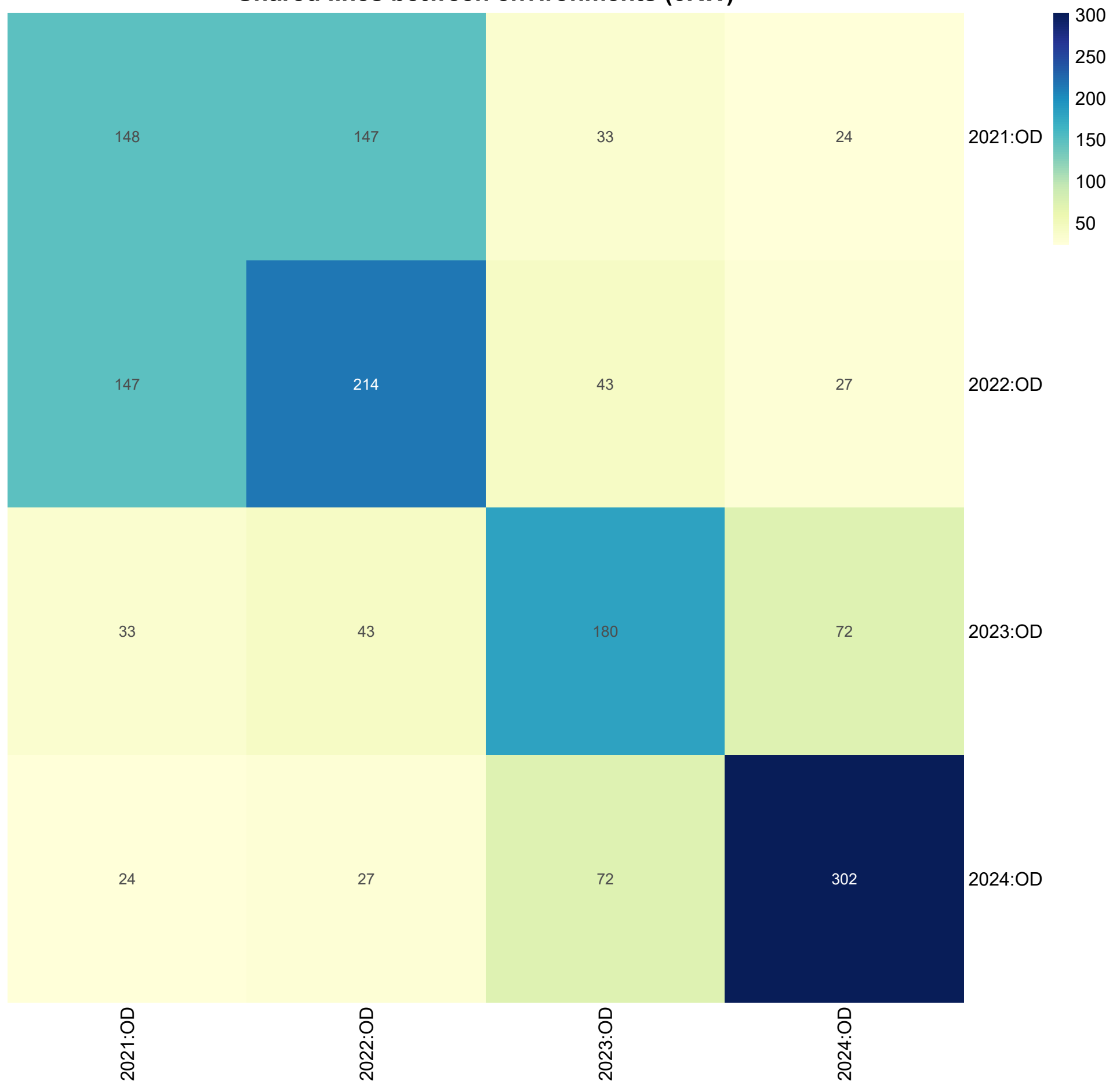

Supplement: jkaf218_Supplementary_Data [file jkaf218_supplementary_data.zip › Figure_S11_G3-2025-406199.pdf]

Shared lines between environments (2RW)

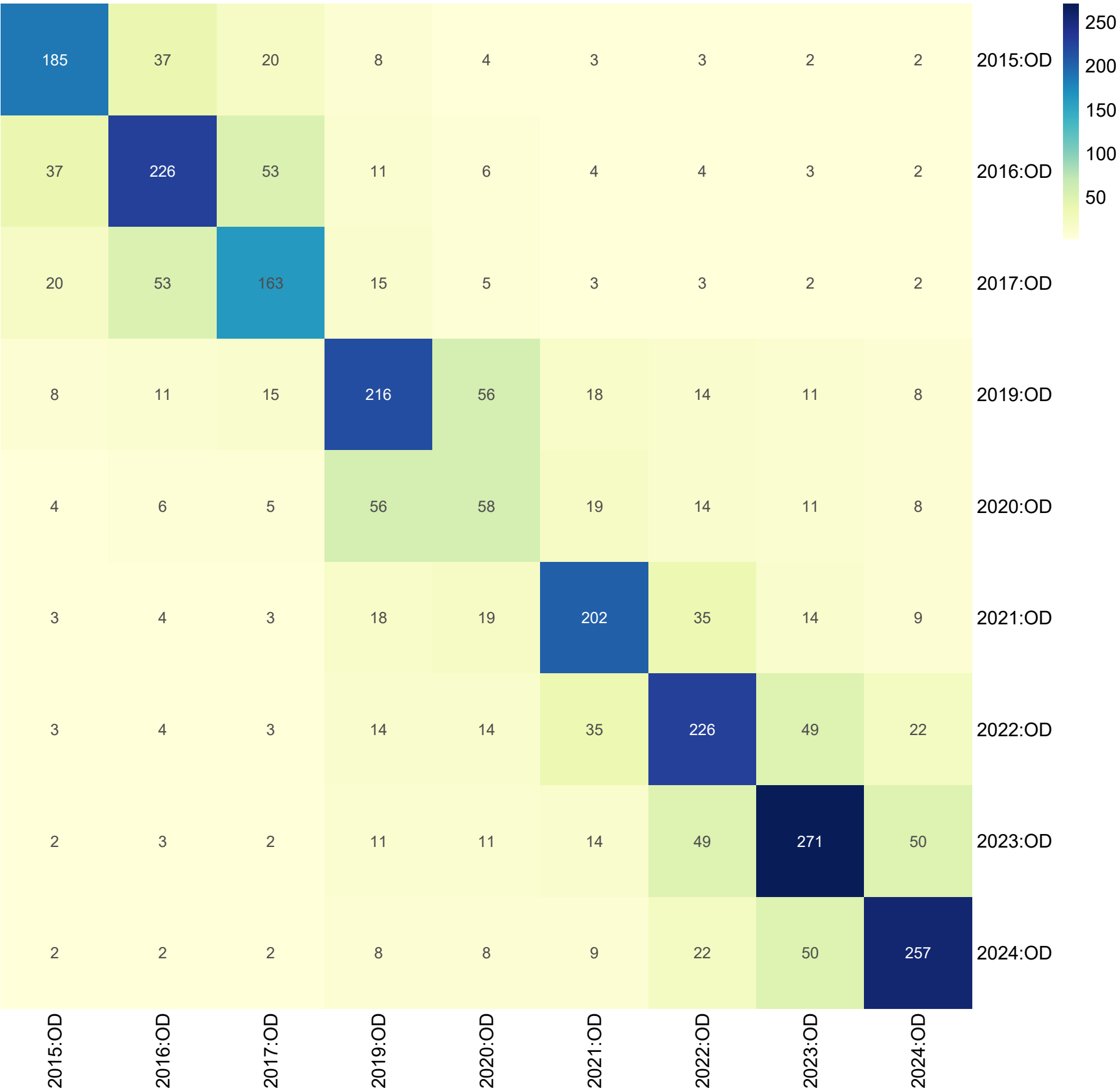

Supplement: jkaf218_Supplementary_Data [file jkaf218_supplementary_data.zip › Figure_S12_G3-2025-406199.pdf]

Shared lines between environments (6RS)

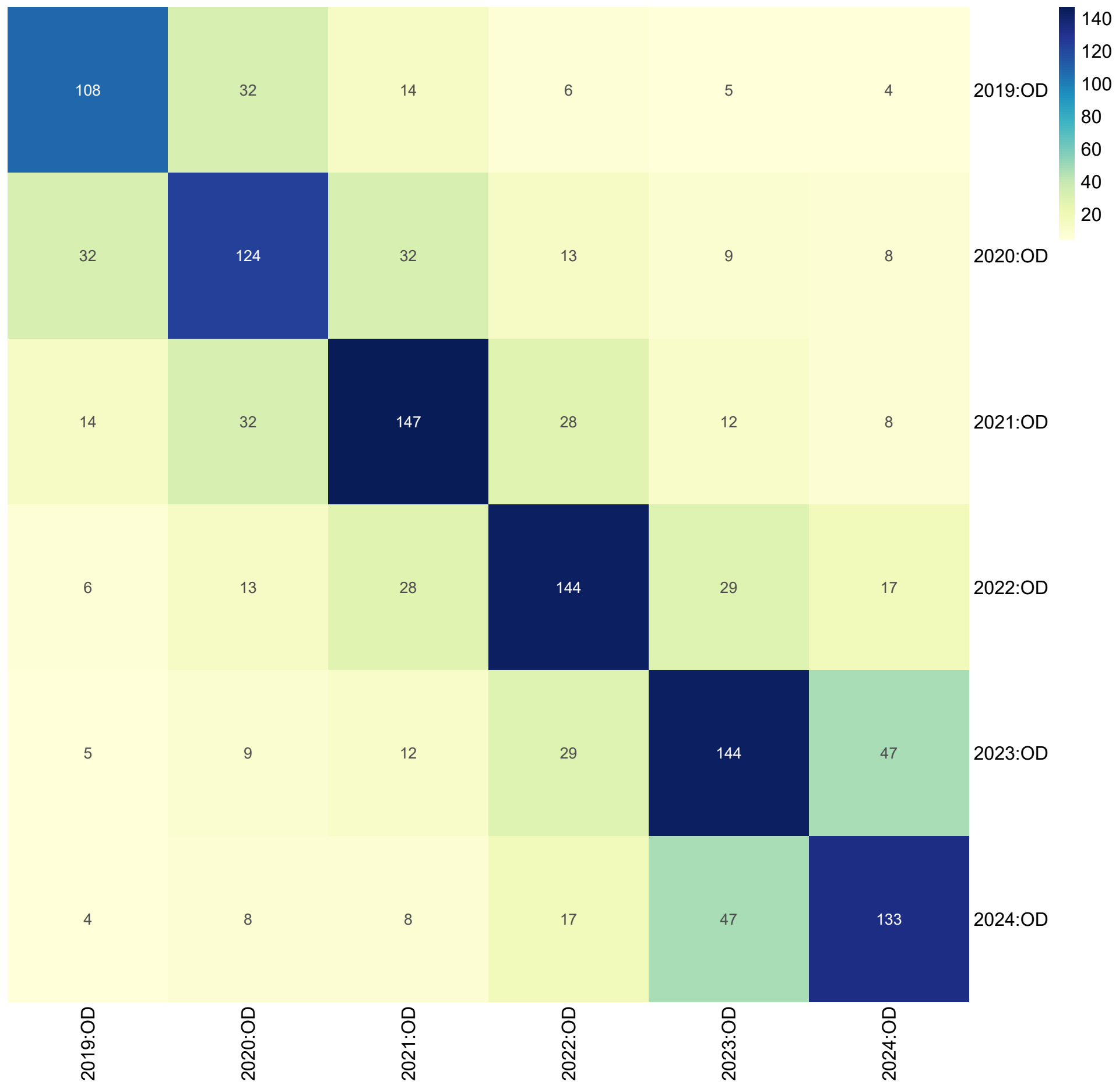

Supplement: jkaf218_Supplementary_Data [file jkaf218_supplementary_data.zip › Figure_S13_G3-2025-406199.pdf]

Shared lines between environments (2RS)

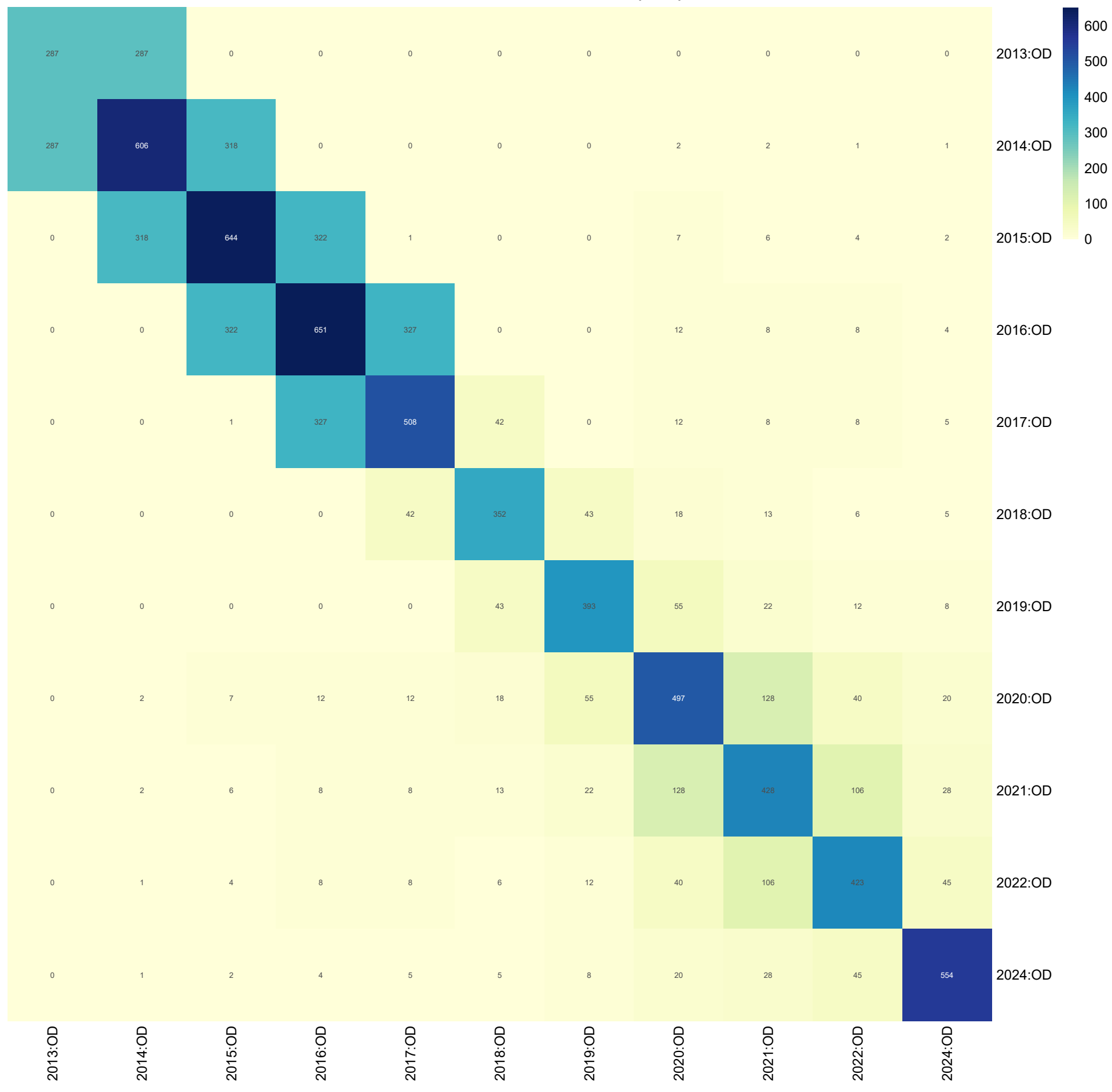

Supplement: jkaf218_Supplementary_Data [file jkaf218_supplementary_data.zip › Figure_S14_G3-2025-406199.pdf]

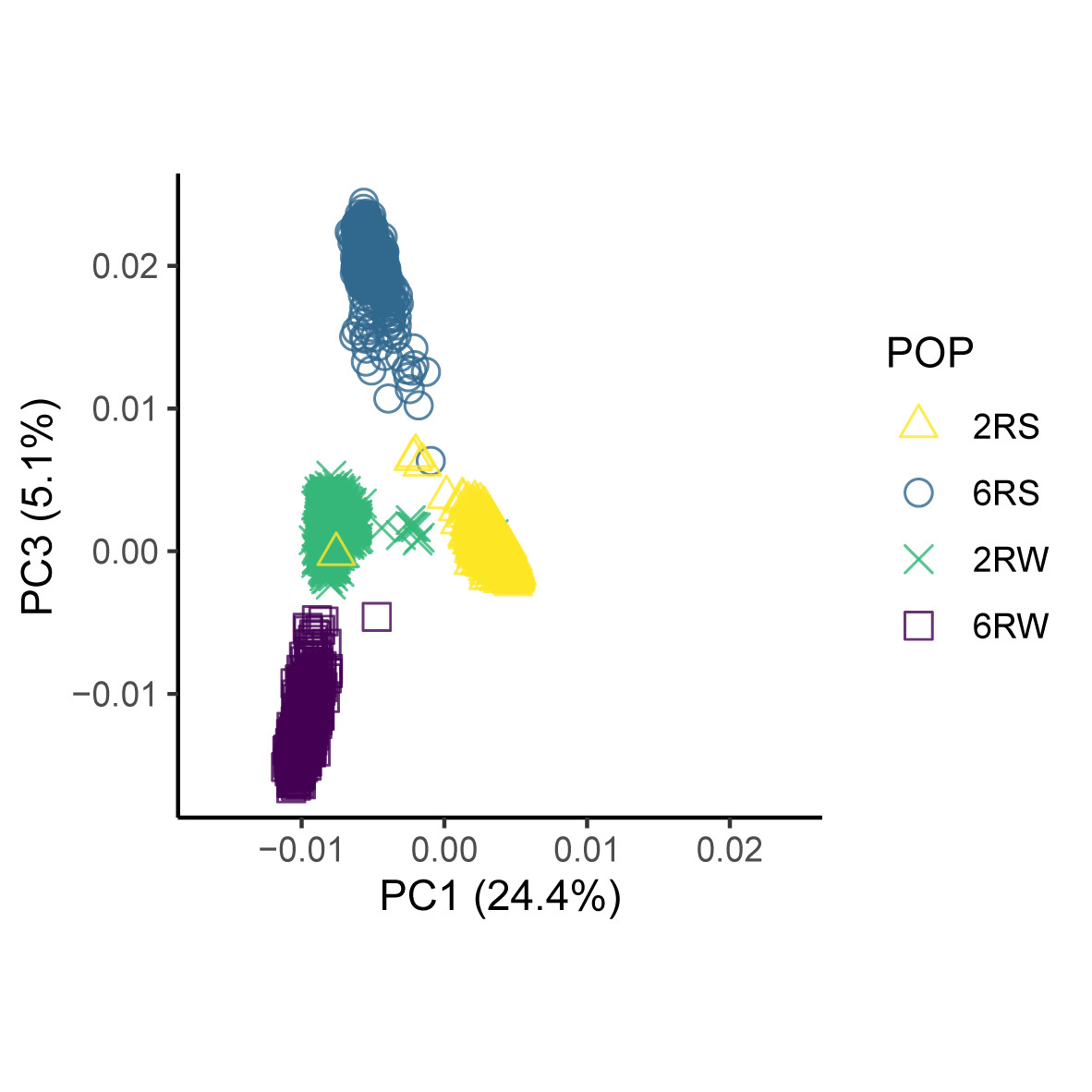

Supplement: jkaf218_Supplementary_Data [file jkaf218_supplementary_data.zip › Figure_S15_G3-2025-406199.png]

# 6RW

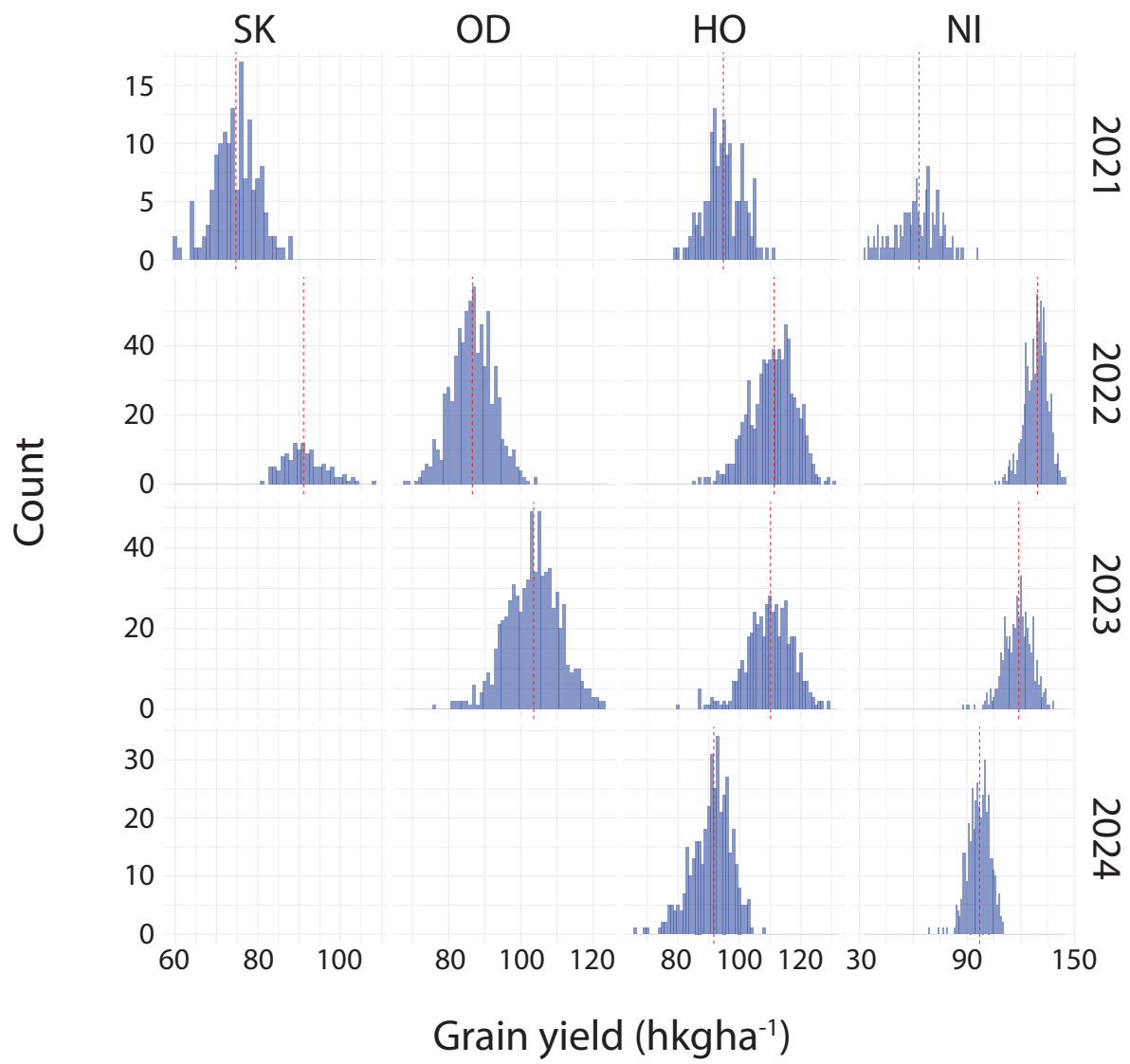

Supplement: jkaf218_Supplementary_Data [file jkaf218_supplementary_data.zip › Figure_S16_G3-2025-406199.pdf]

# 2RW

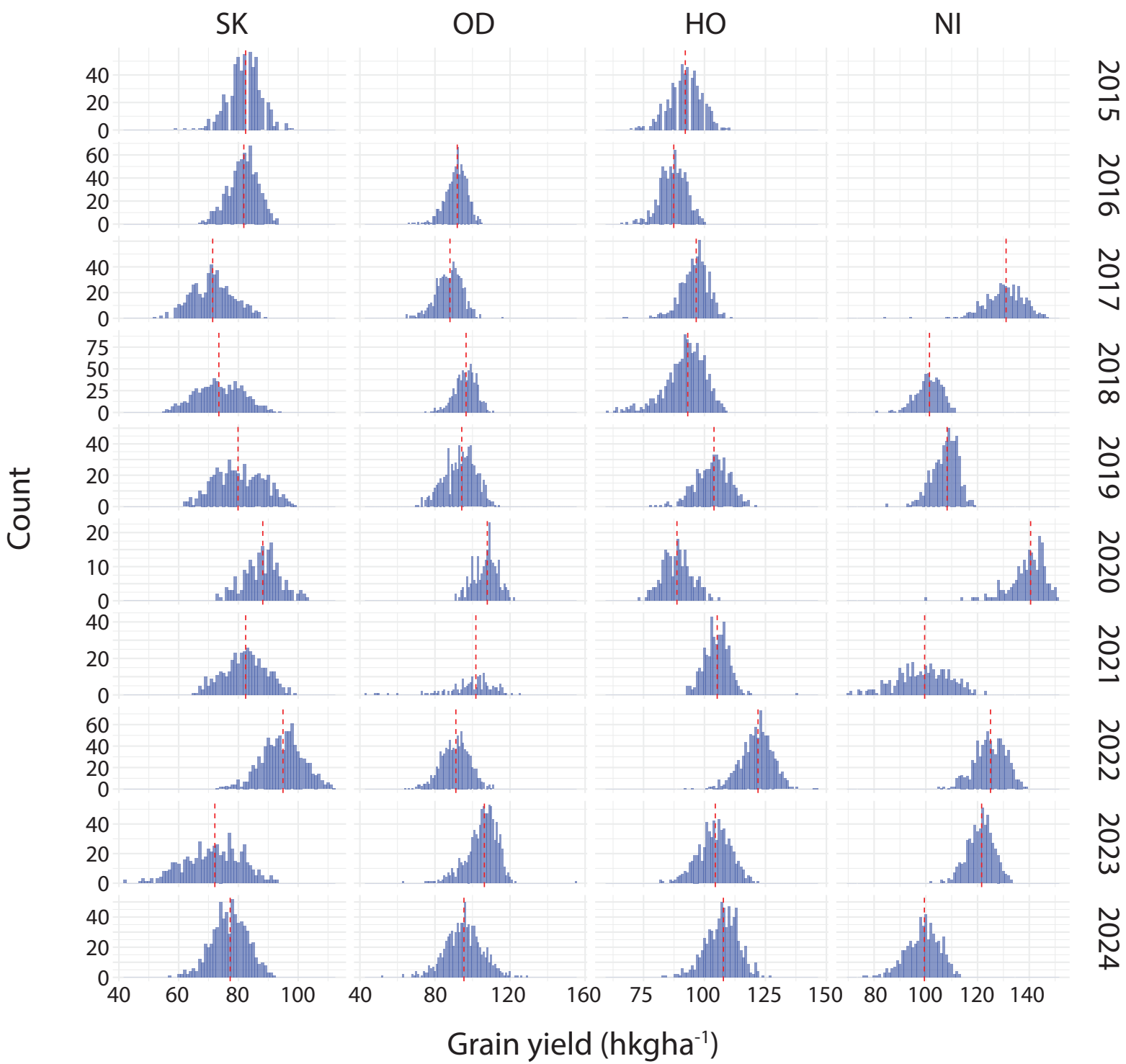

Supplement: jkaf218_Supplementary_Data [file jkaf218_supplementary_data.zip › Figure_S17_G3-2025-406199.pdf]

# 6RS

OD

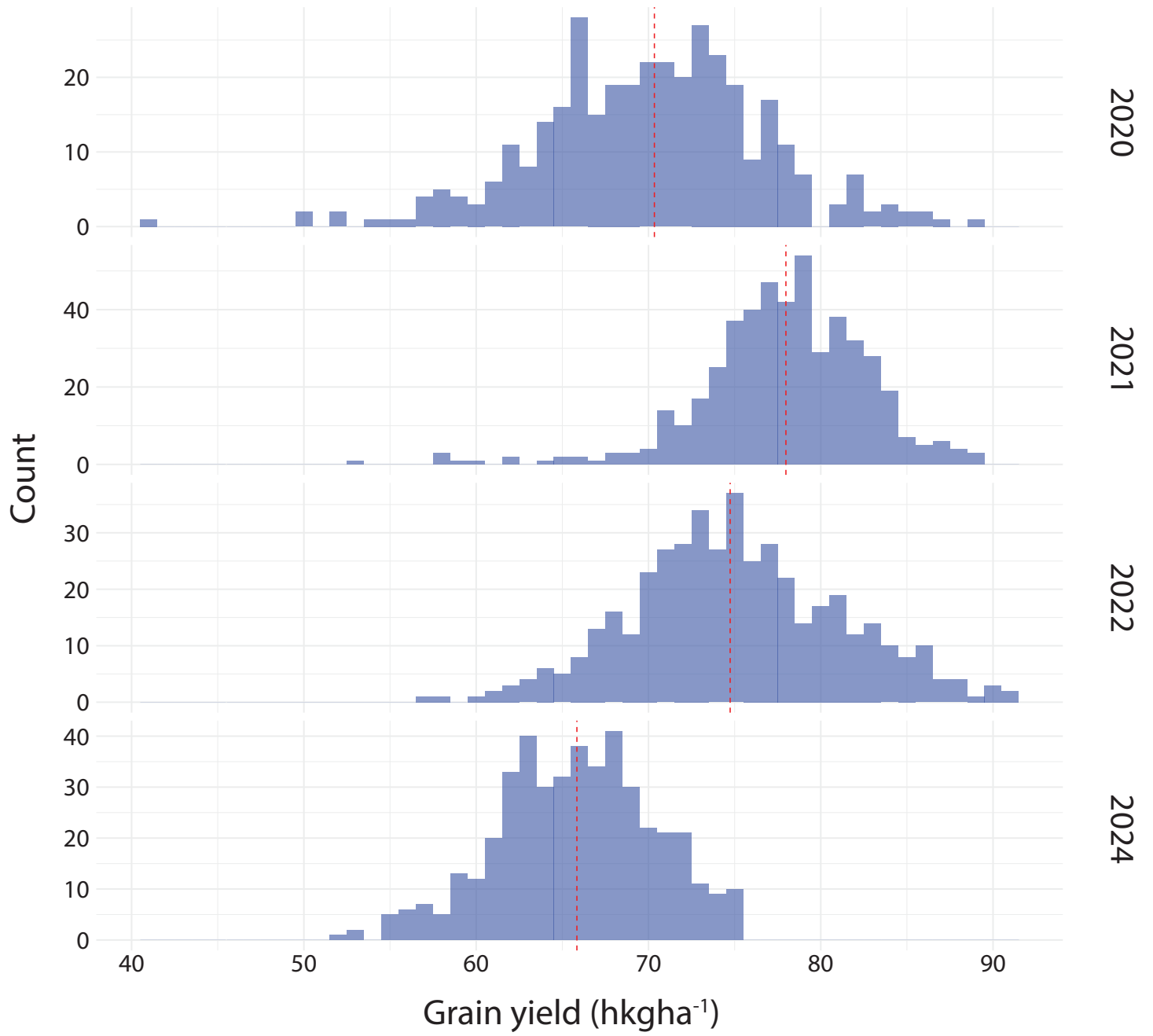

Supplement: jkaf218_Supplementary_Data [file jkaf218_supplementary_data.zip › Figure_S18_G3-2025-406199.pdf]

2RS

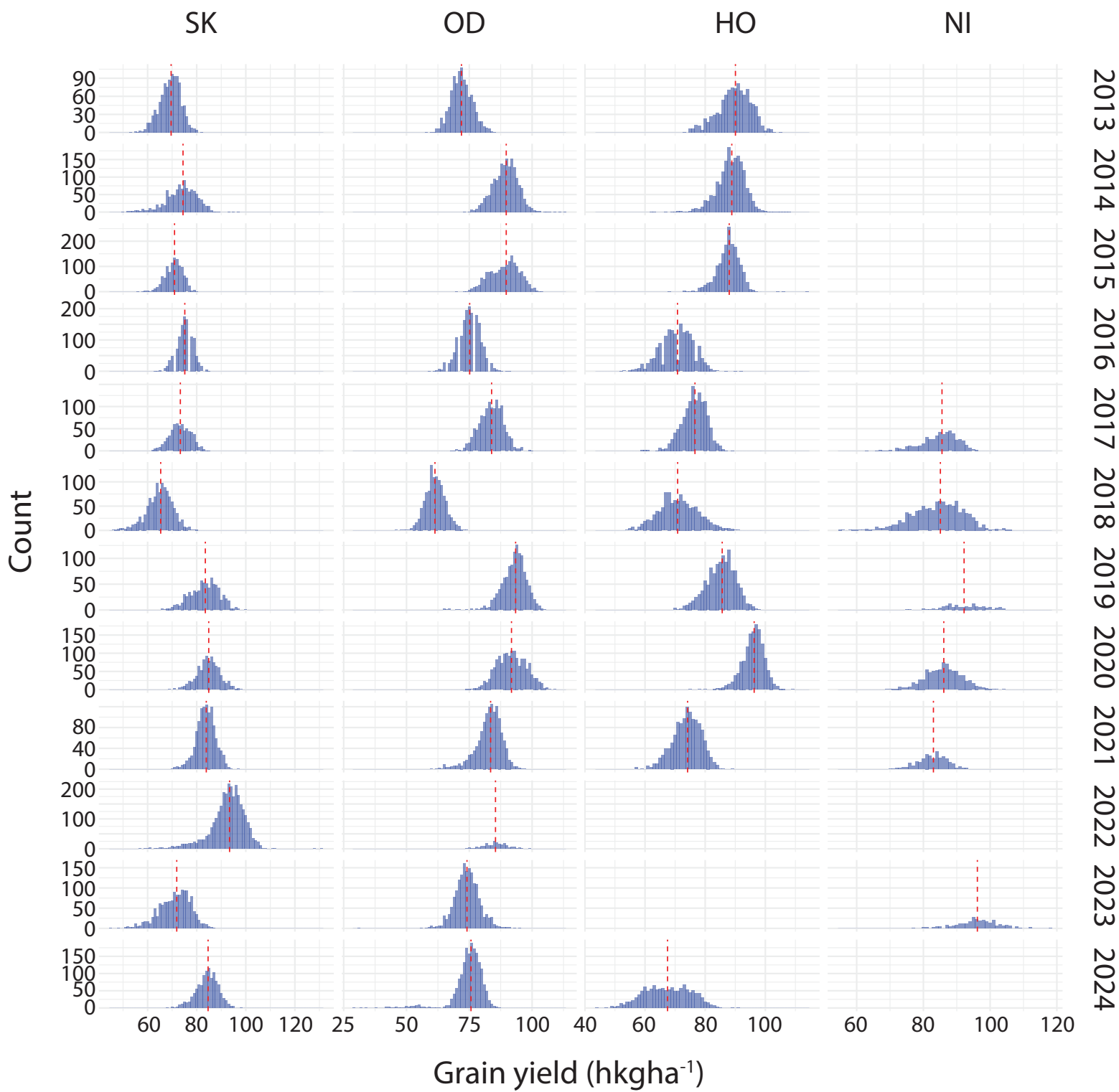

Supplement: jkaf218_Supplementary_Data [file jkaf218_supplementary_data.zip › Figure_S19_G3-2025-406199.pdf]

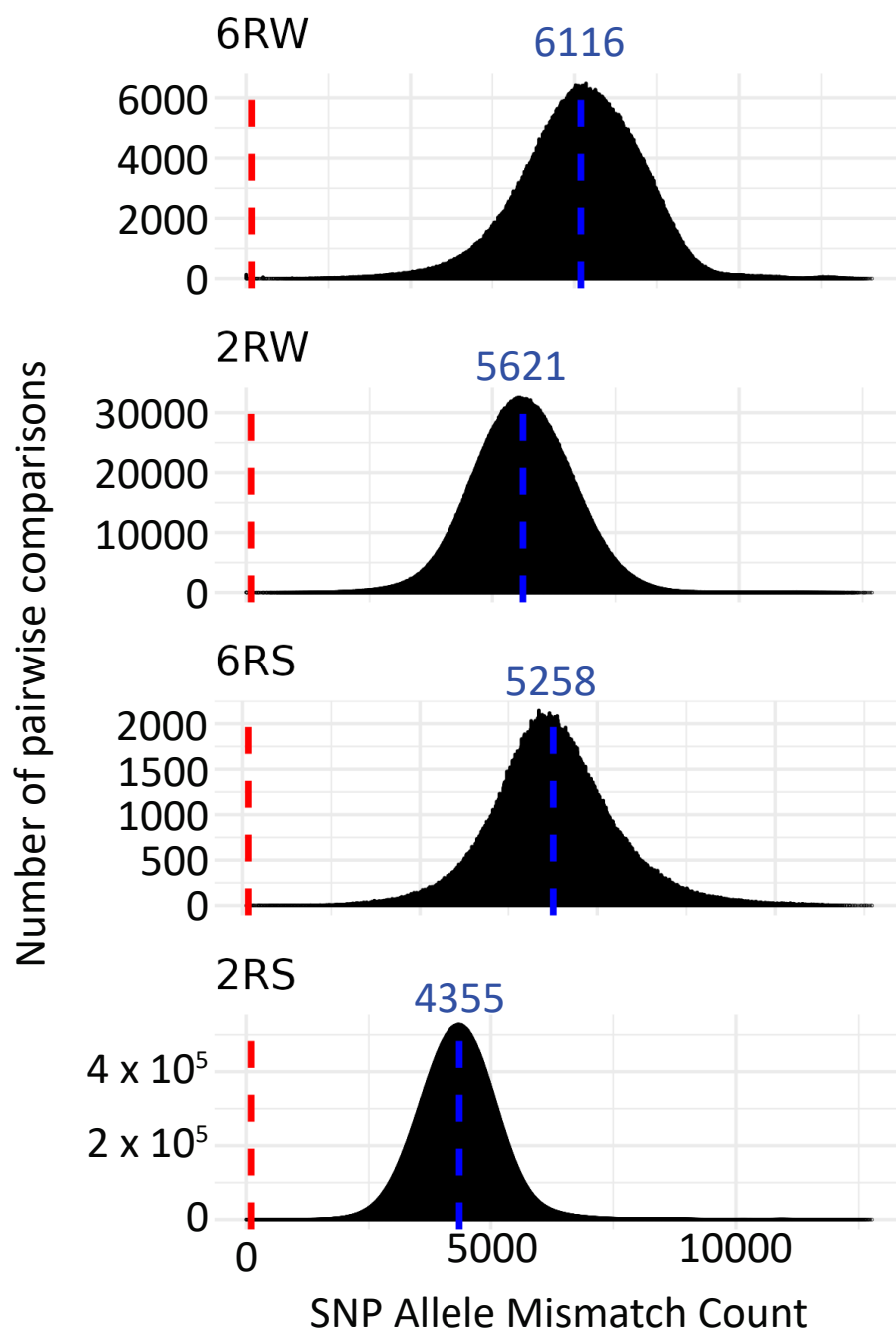

Supplement: jkaf218_Supplementary_Data [file jkaf218_supplementary_data.zip › Figure_S2_G3-2025-406199.pdf]

6RW

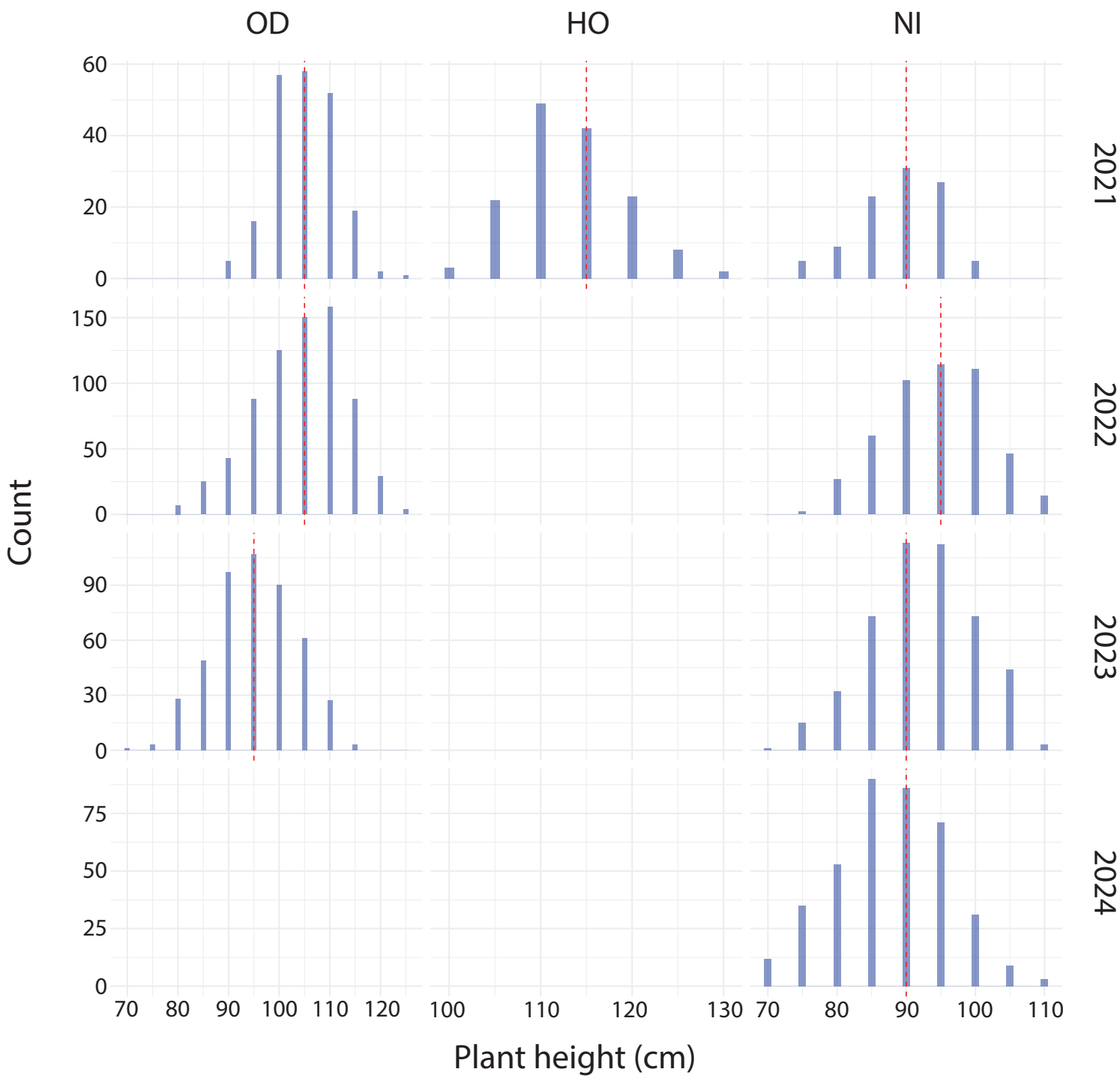

Supplement: jkaf218_Supplementary_Data [file jkaf218_supplementary_data.zip › Figure_S20_G3-2025-406199.pdf]

2RW

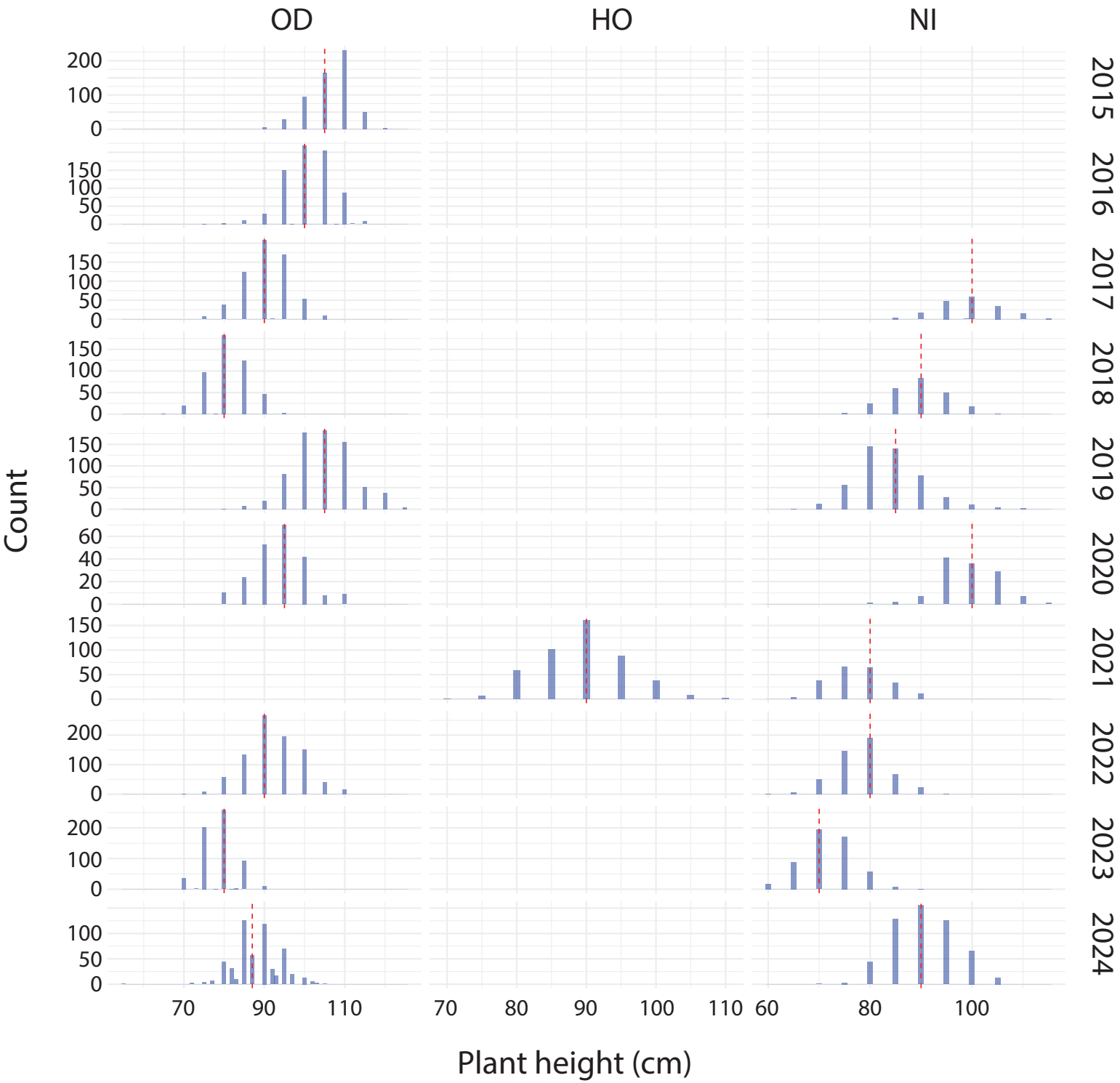

Supplement: jkaf218_Supplementary_Data [file jkaf218_supplementary_data.zip › Figure_S21_G3-2025-406199.pdf]

6RS

OD

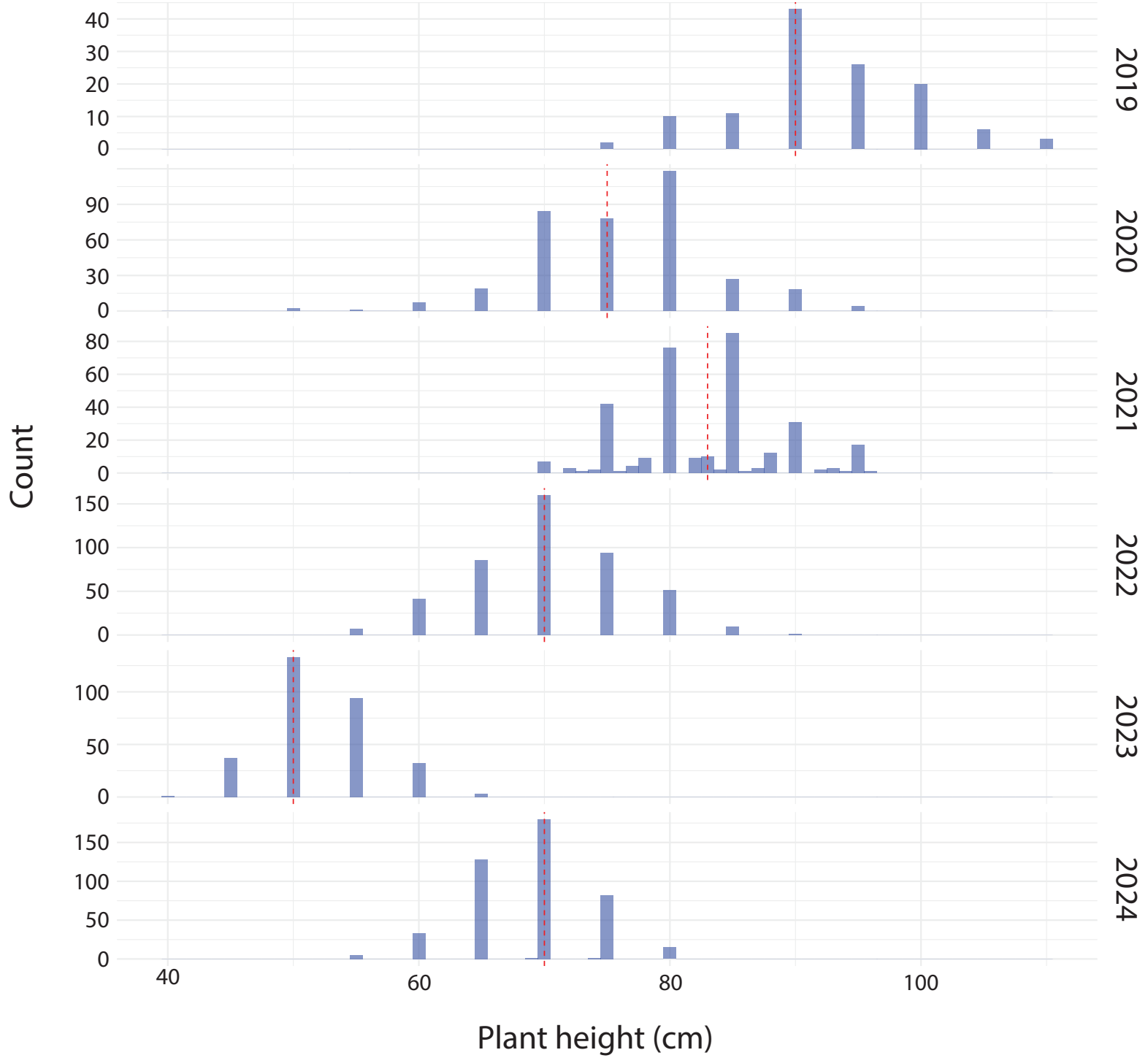

Supplement: jkaf218_Supplementary_Data [file jkaf218_supplementary_data.zip › Figure_S22_G3-2025-406199.pdf]

# 2RS

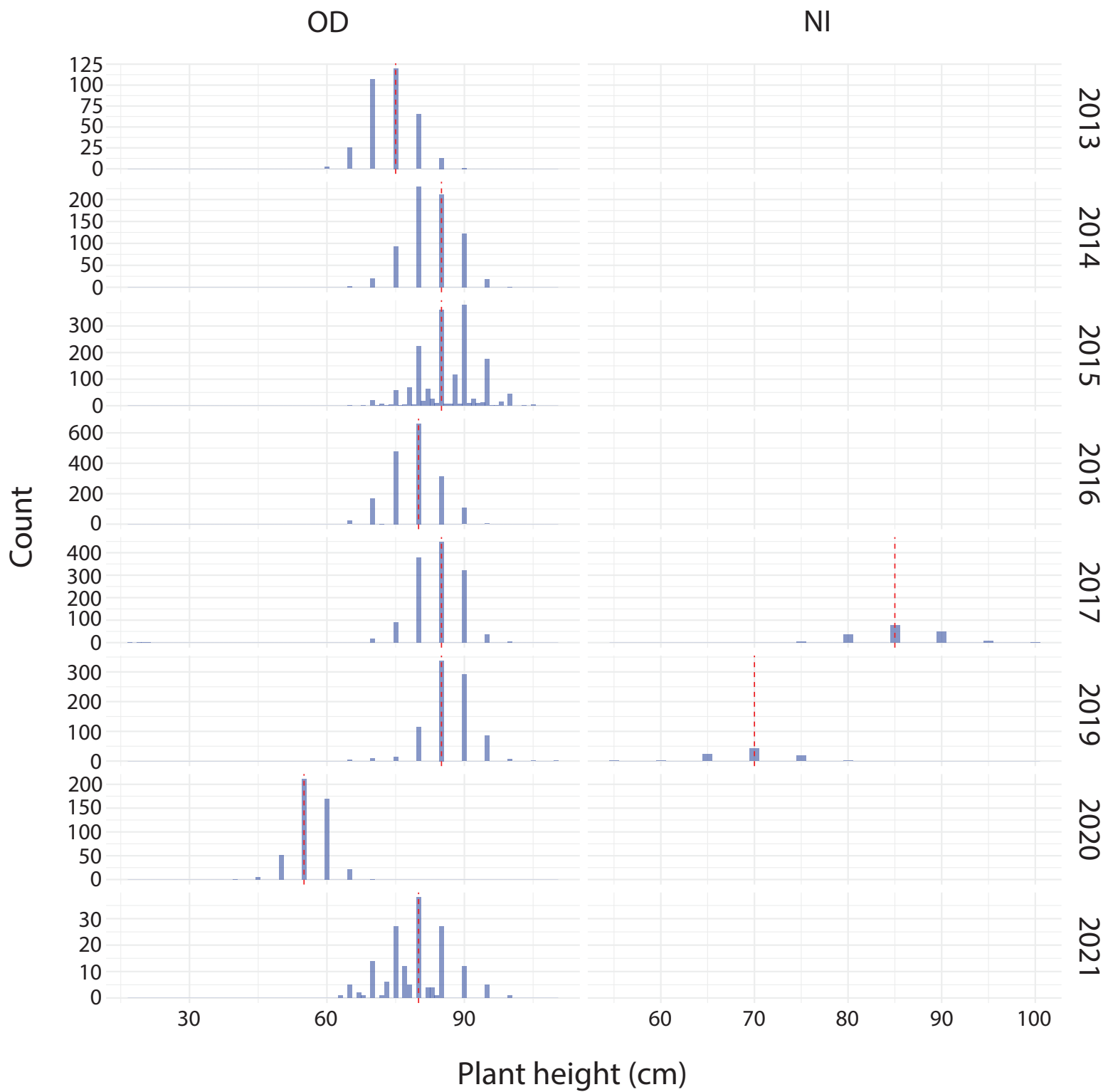

Supplement: jkaf218_Supplementary_Data [file jkaf218_supplementary_data.zip › Figure_S23_G3-2025-406199.pdf]

6RW

OD

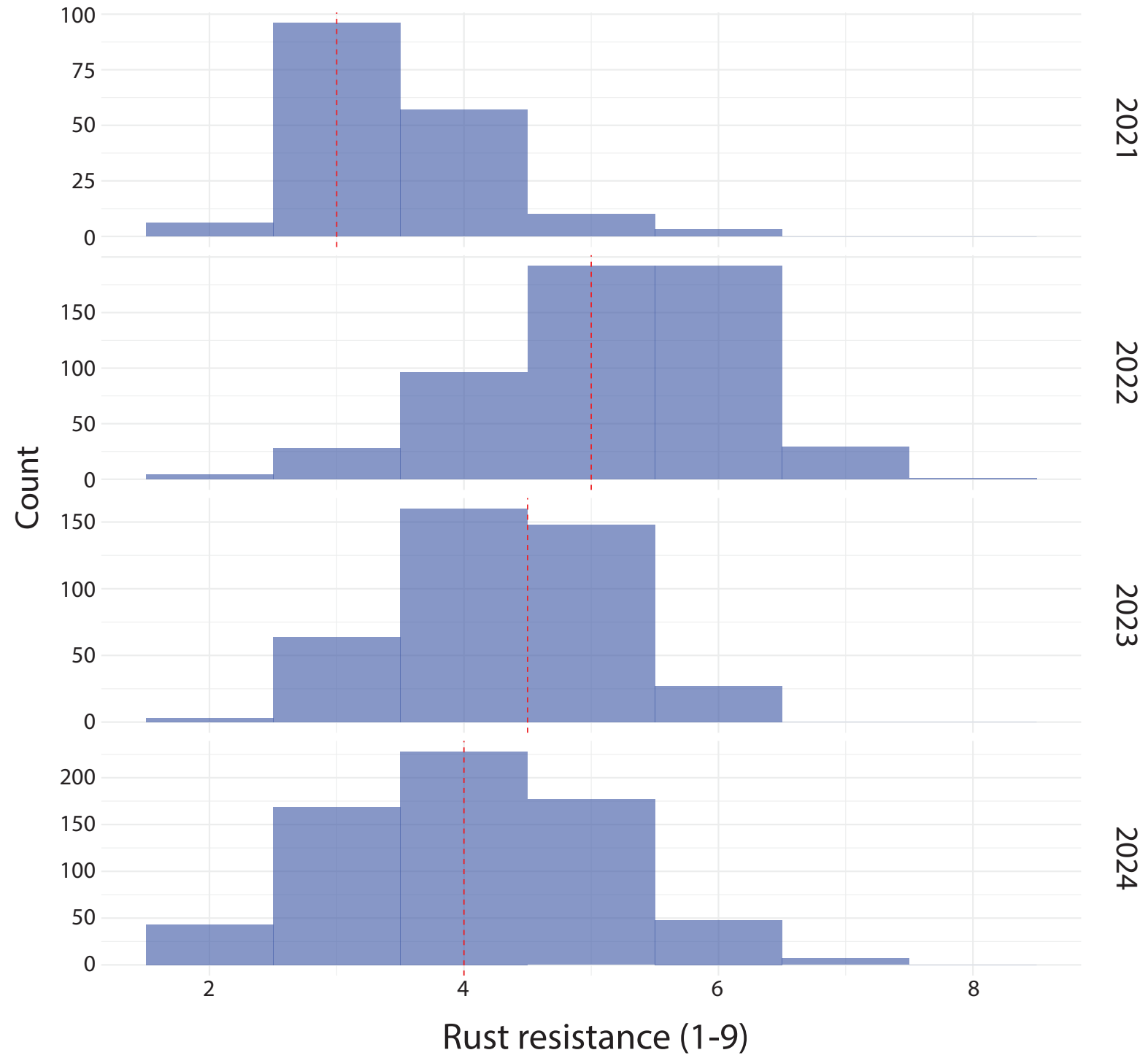

Supplement: jkaf218_Supplementary_Data [file jkaf218_supplementary_data.zip › Figure_S24_G3-2025-406199.pdf]

2RW

OD

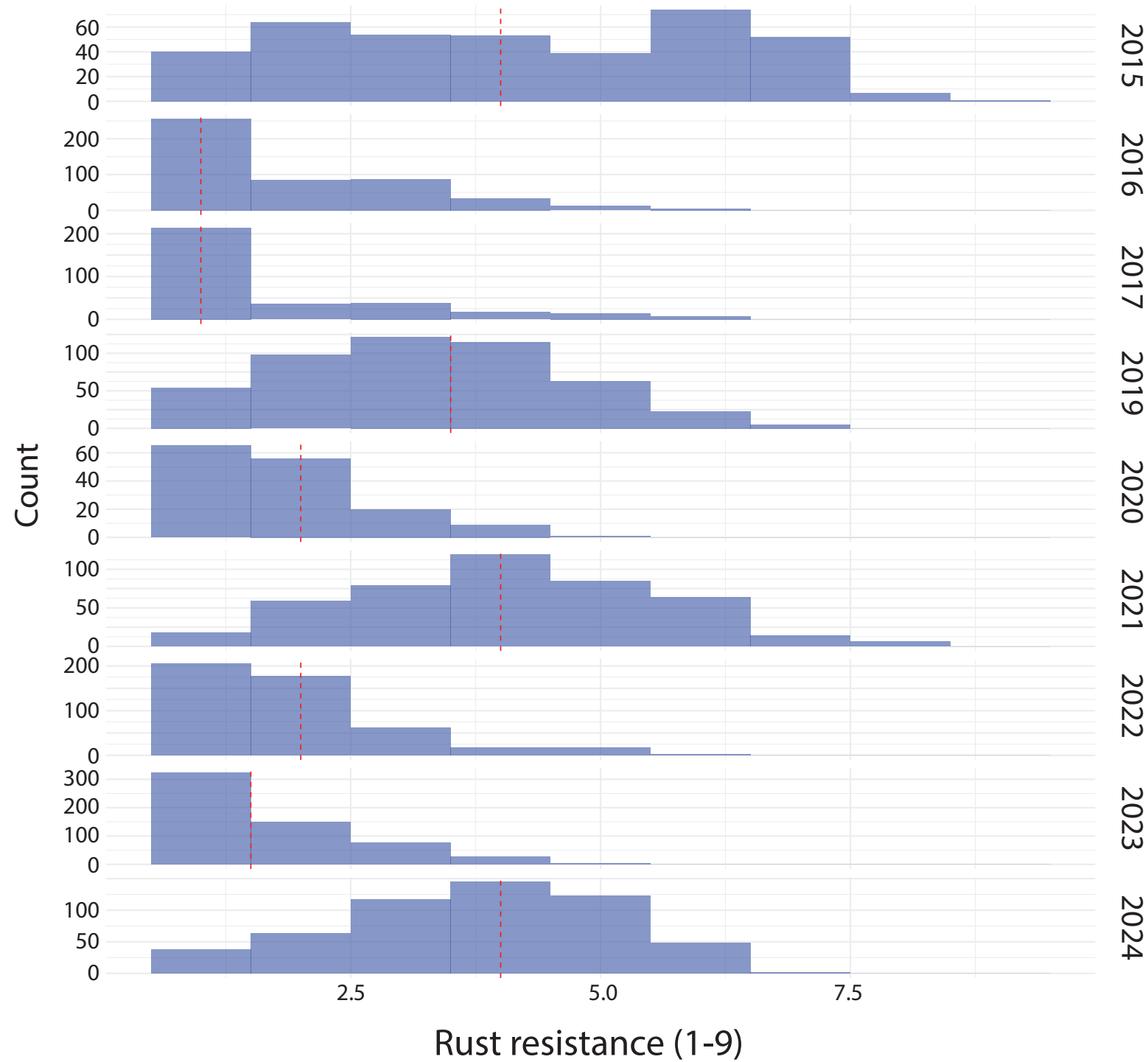

Supplement: jkaf218_Supplementary_Data [file jkaf218_supplementary_data.zip › Figure_S25_G3-2025-406199.pdf]

6RS

OD

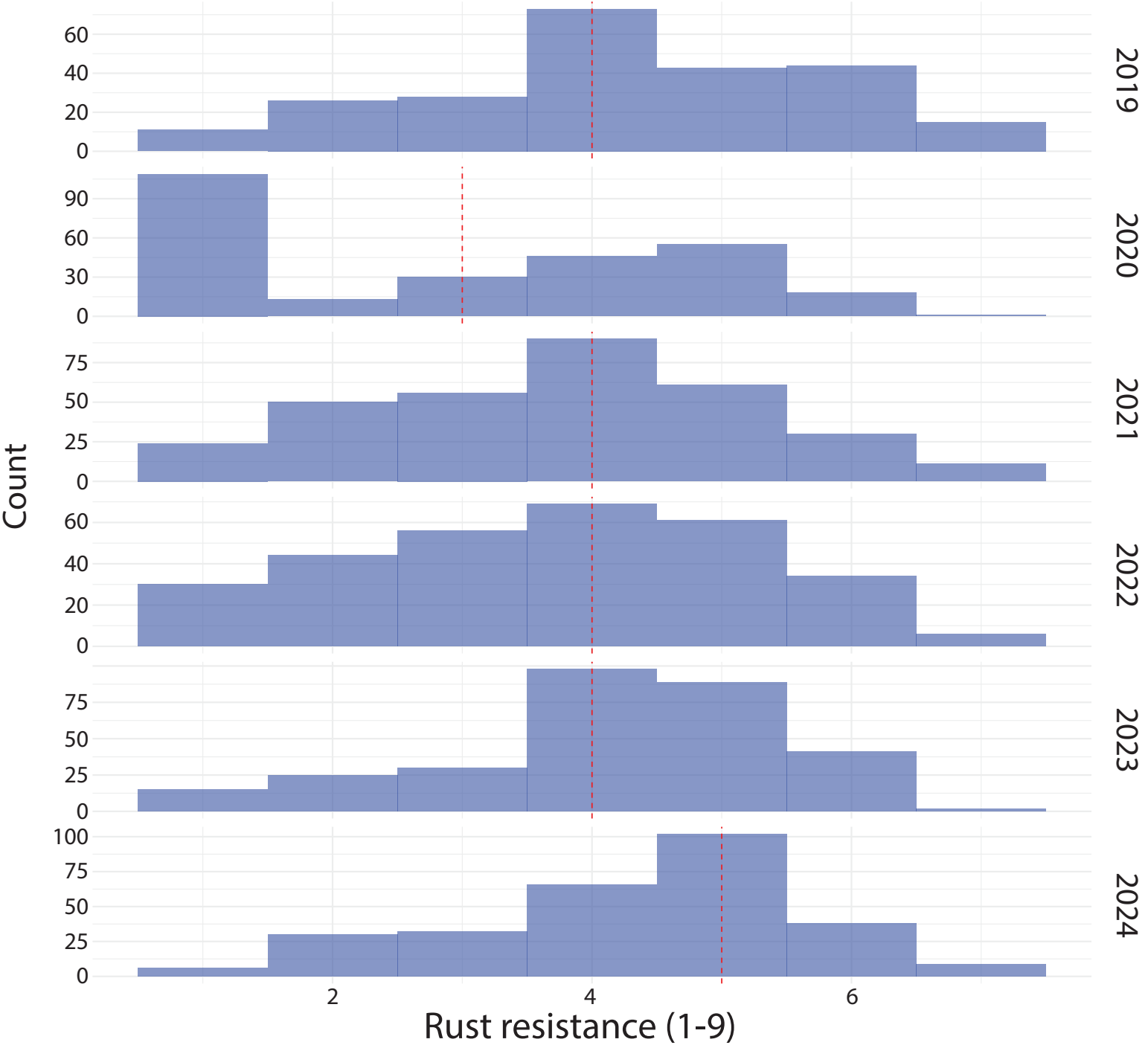

Supplement: jkaf218_Supplementary_Data [file jkaf218_supplementary_data.zip › Figure_S26_G3-2025-406199.pdf]

2RS

OD

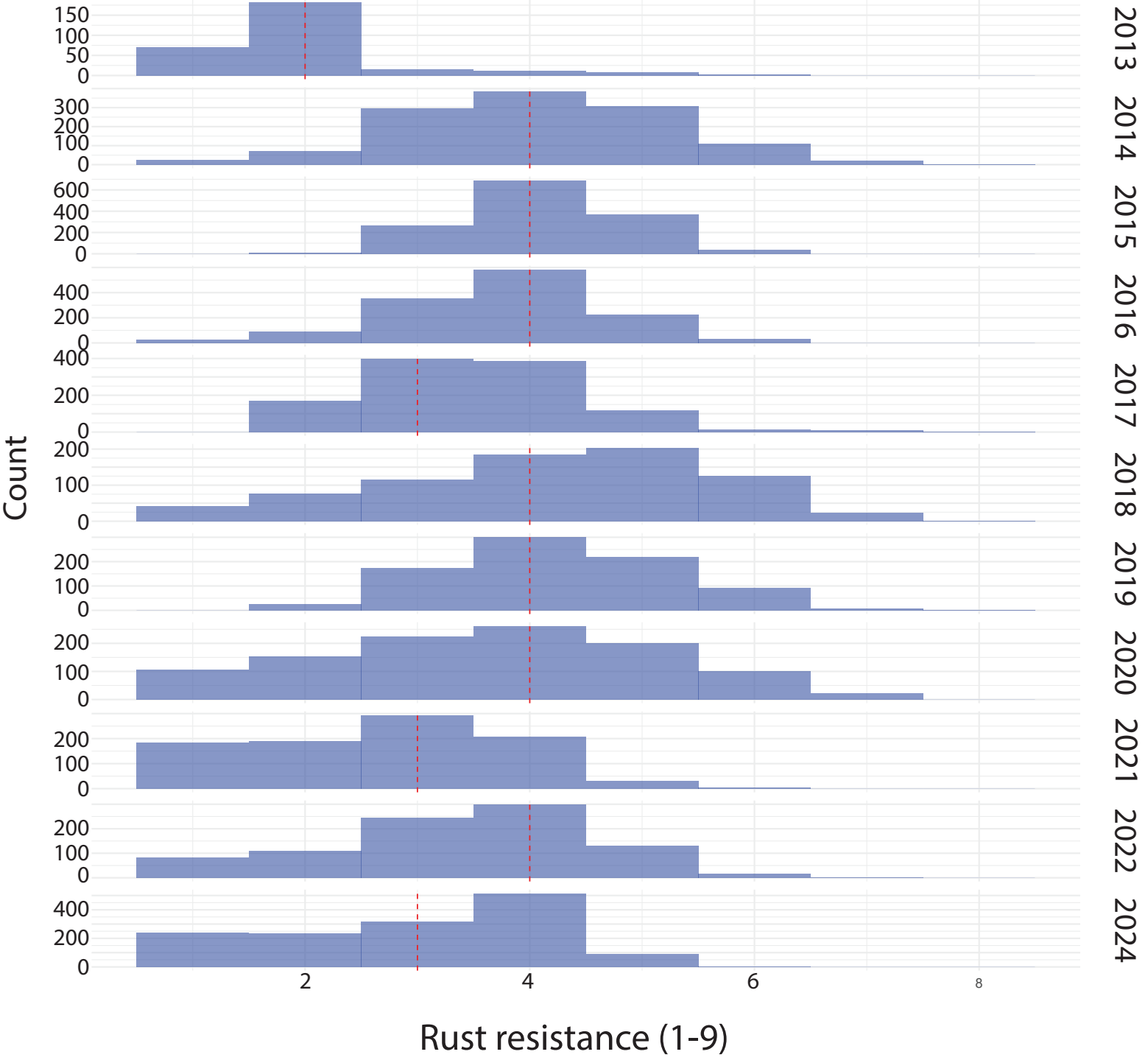

Supplement: jkaf218_Supplementary_Data [file jkaf218_supplementary_data.zip › Figure_S27_G3-2025-406199.pdf]

Fraction of families represented

0.00

0.25

0.50

0.75

0

100

200

300

400

Size of training population

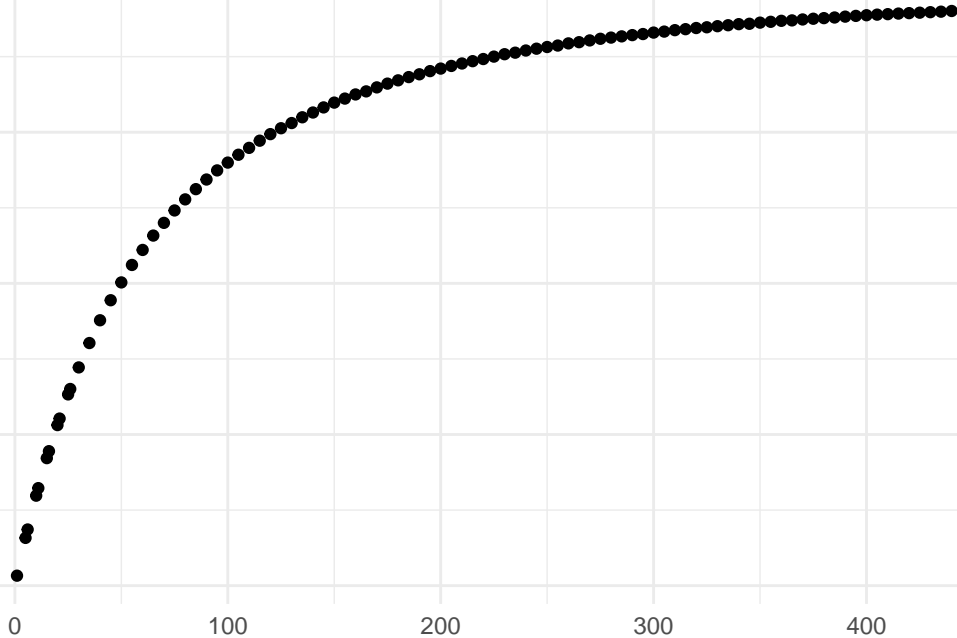

Supplement: jkaf218_Supplementary_Data [file jkaf218_supplementary_data.zip › Figure_S29_G3-2025-406199.pdf]

Shared lines between environments (6RW)

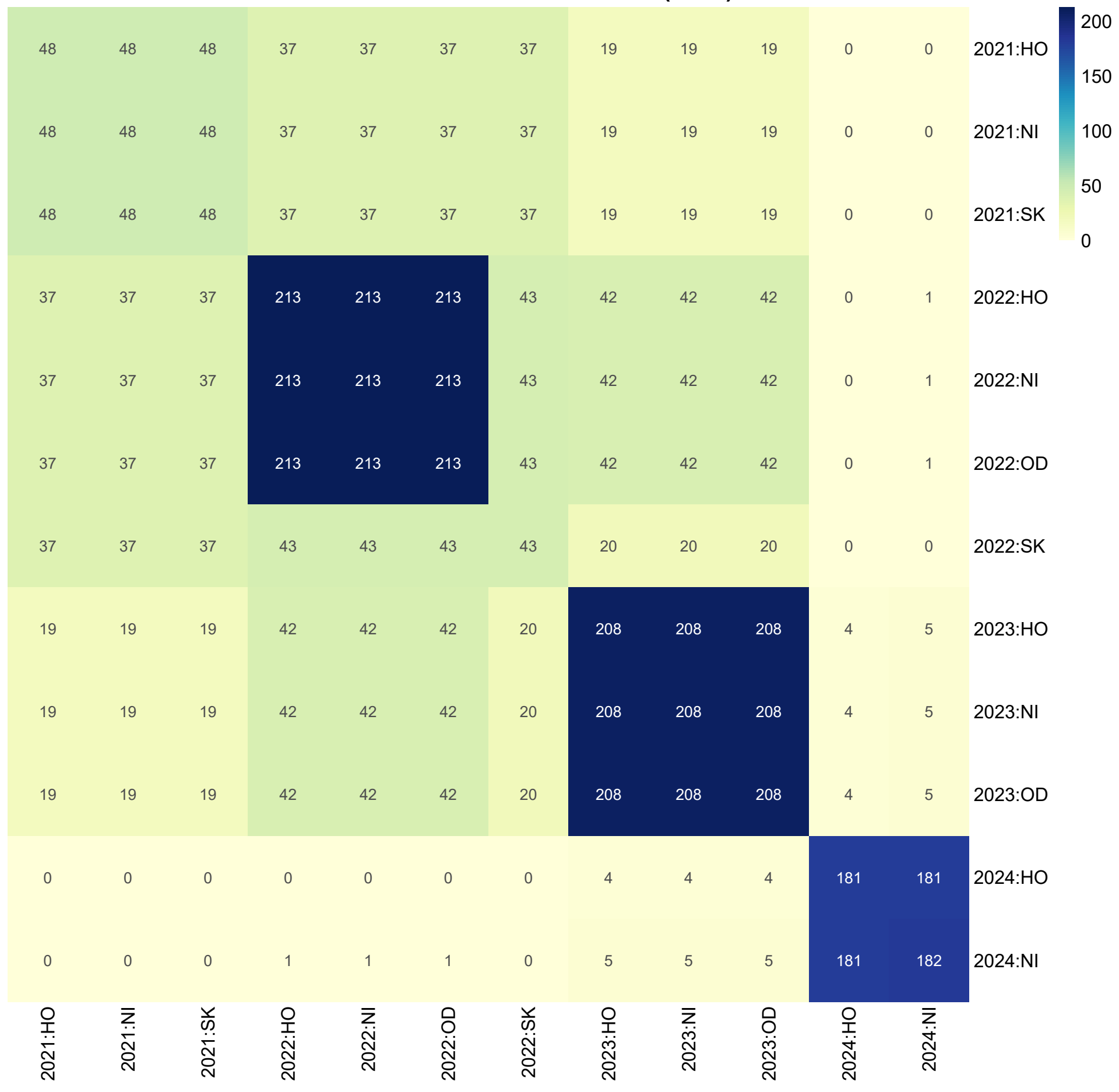

Supplement: jkaf218_Supplementary_Data [file jkaf218_supplementary_data.zip › Figure_S3_G3-2025-406199.pdf]

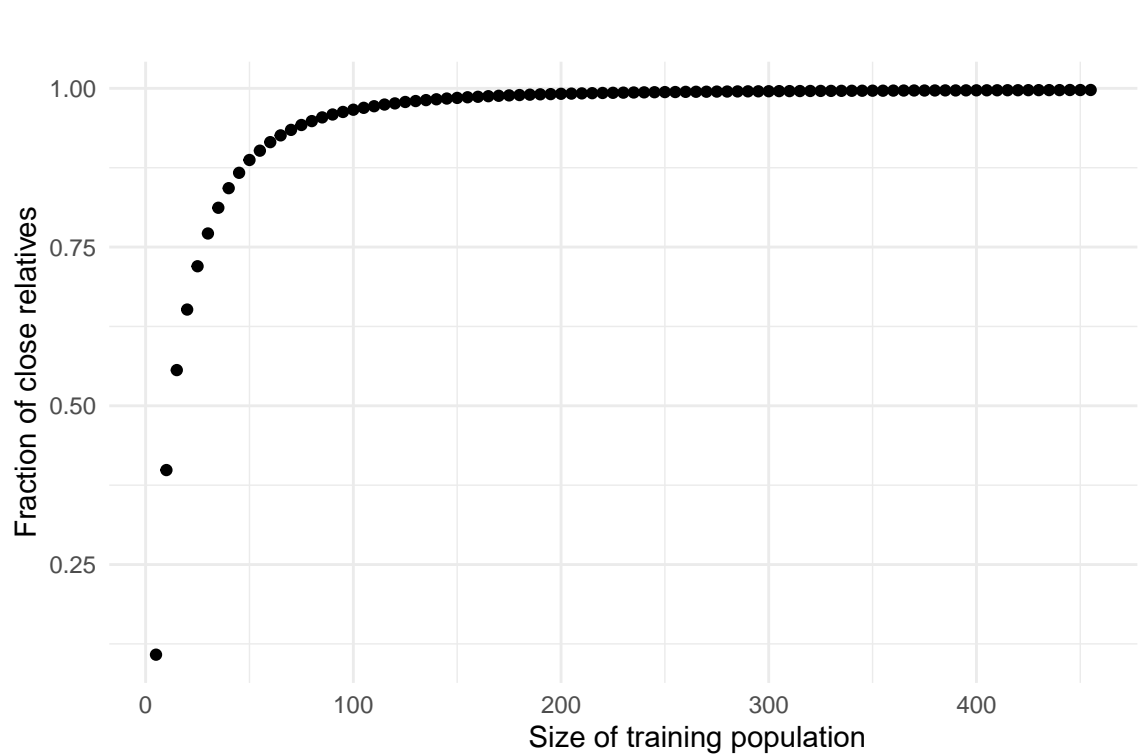

Supplement: jkaf218_Supplementary_Data [file jkaf218_supplementary_data.zip › Figure_S30_G3-2025-406199.pdf]

Shared lines between environments (6RS)

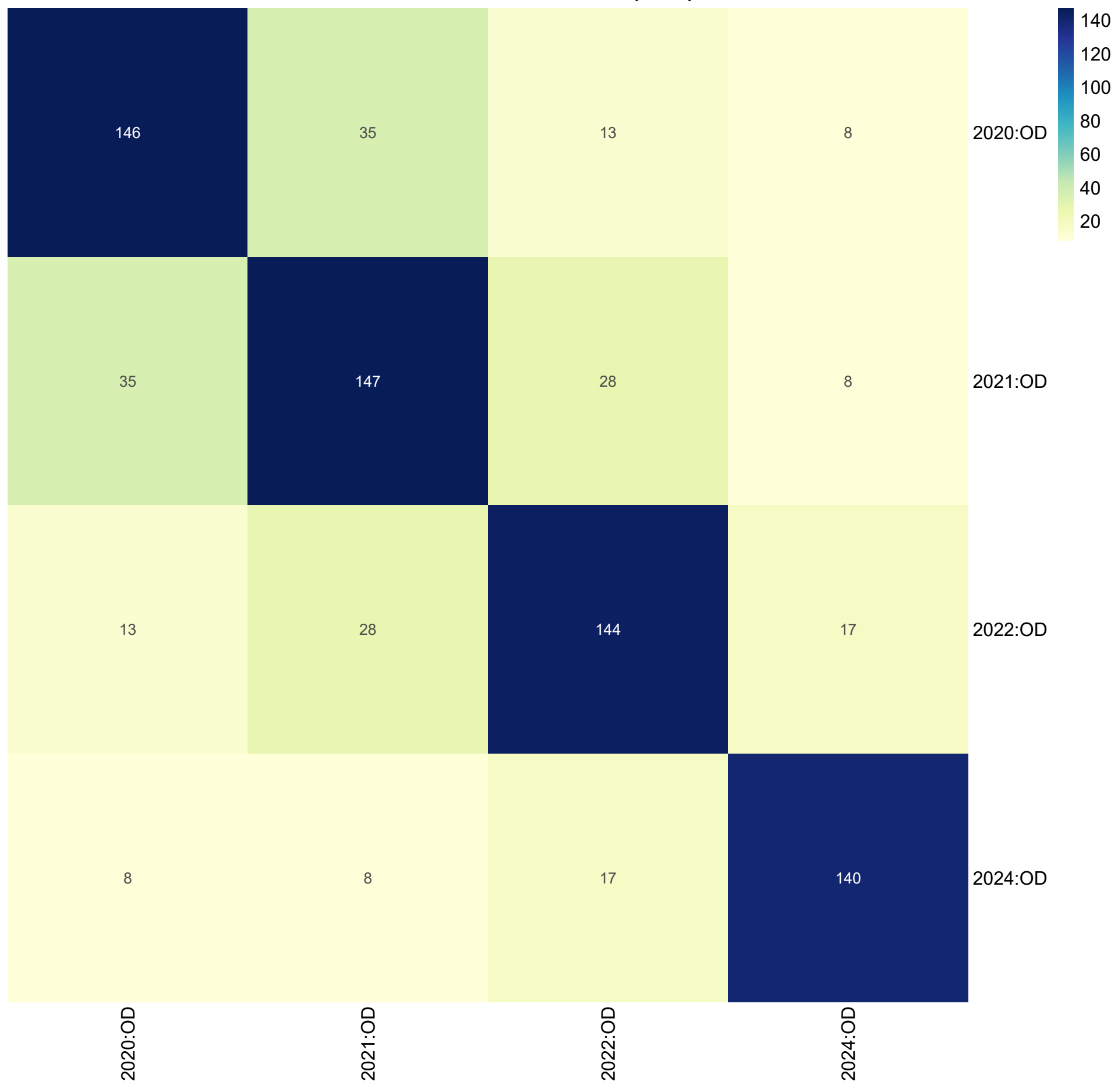

Supplement: jkaf218_Supplementary_Data [file jkaf218_supplementary_data.zip › Figure_S5_G3-2025-406199.pdf]

### Shared lines between environments (2RS)

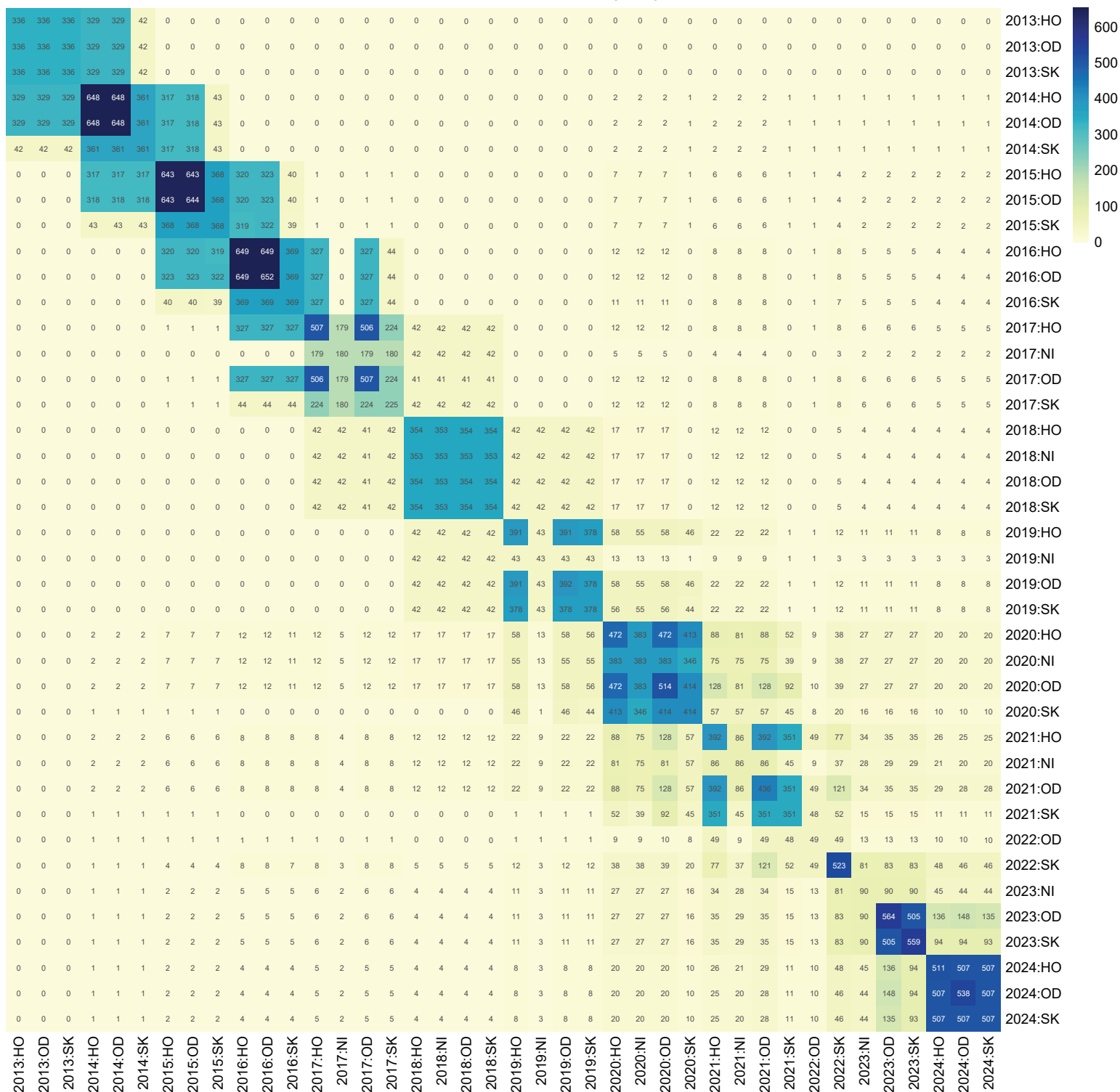

Supplement: jkaf218_Supplementary_Data [file jkaf218_supplementary_data.zip › Figure_S6_G3-2025-406199.pdf]

Shared lines between environments (6RW)

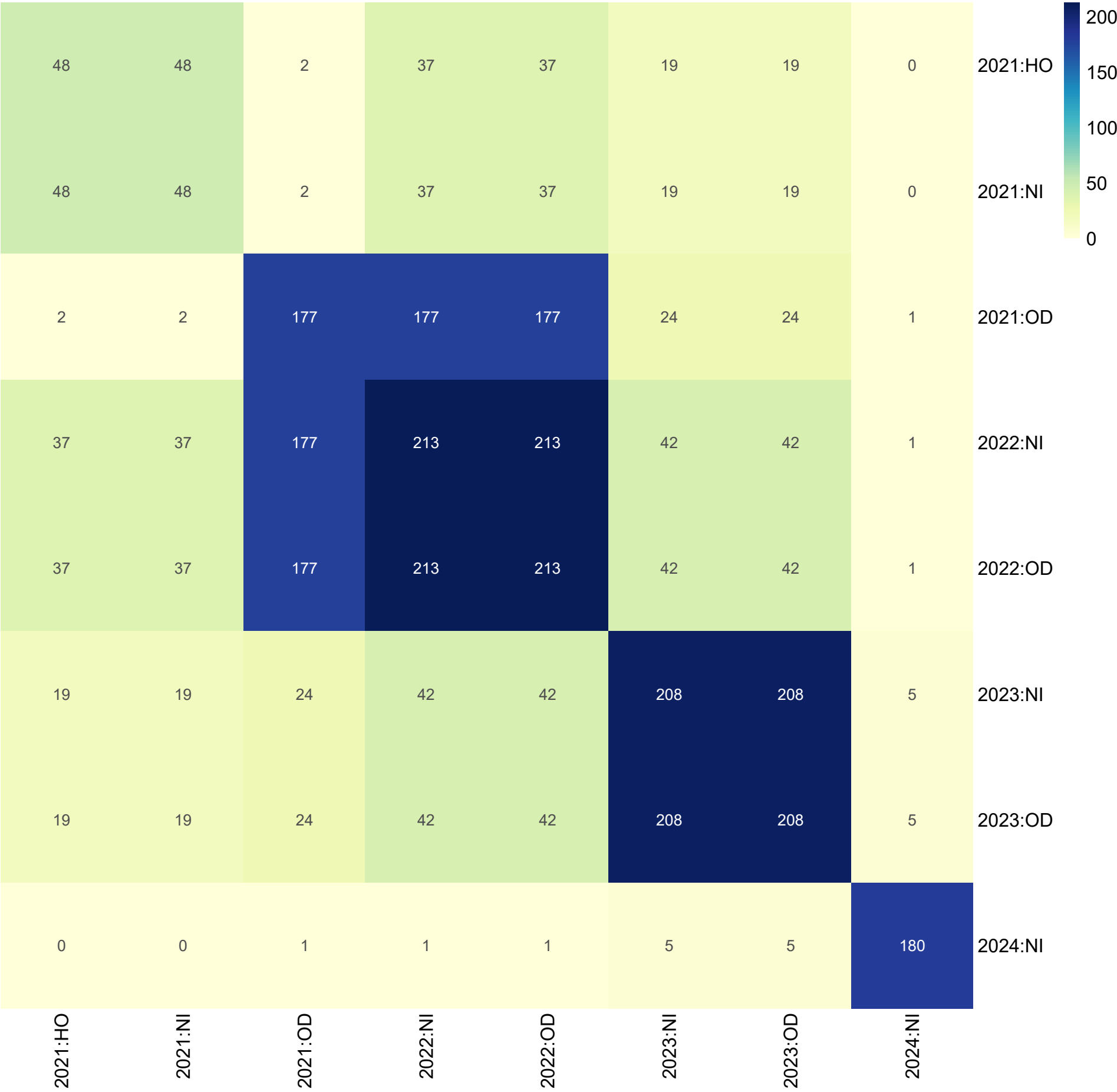

Supplement: jkaf218_Supplementary_Data [file jkaf218_supplementary_data.zip › Figure_S7_G3-2025-406199.pdf]

Shared lines between environments (2RW)

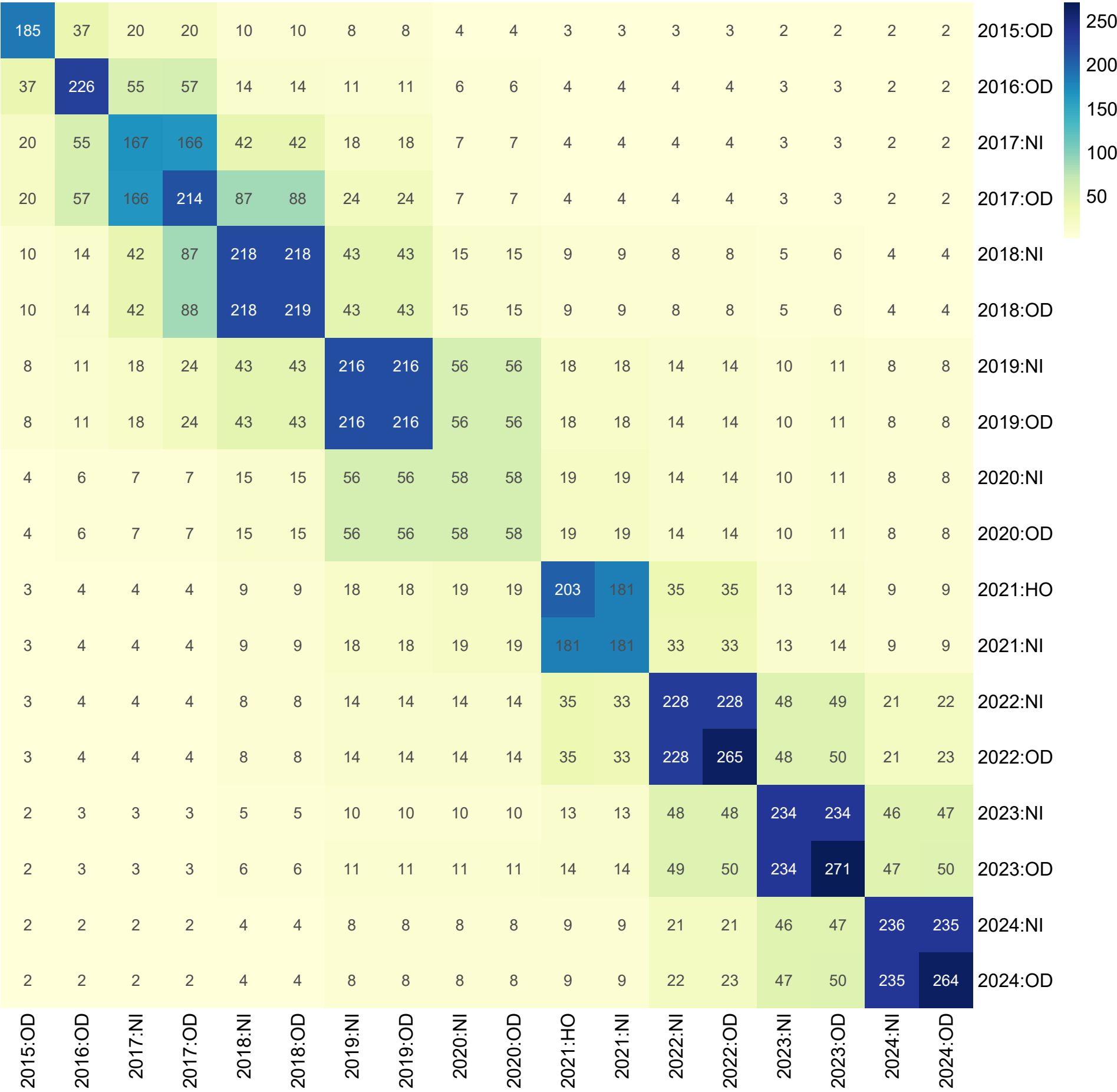

Supplement: jkaf218_Supplementary_Data [file jkaf218_supplementary_data.zip › Figure_S8_G3-2025-406199.pdf]

Shared lines between environments (6RS)

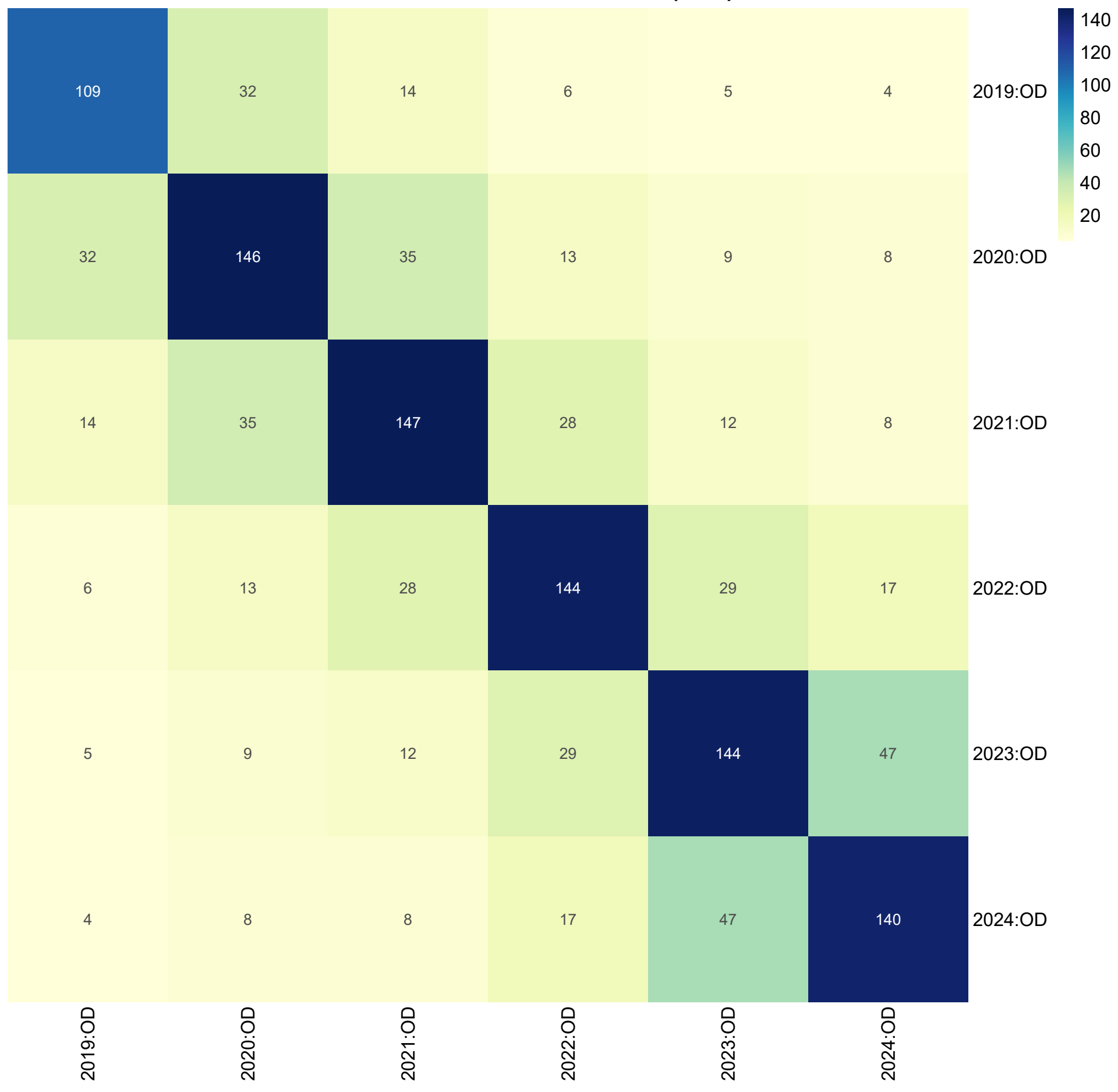

Supplement: jkaf218_Supplementary_Data [file jkaf218_supplementary_data.zip › Figure_S9_G3-2025-406199.pdf]
